# IGF1 Knockdown Hinders Myocardial Development through Energy Metabolism Dysfunction Caused by ROS-Dependent FOXO Activation in the Chicken Heart

**DOI:** 10.1155/2019/7838754

**Published:** 2019-12-24

**Authors:** Yafan Gong, Jie Yang, Qi Liu, Jingzeng Cai, Yingying Zheng, Yuan Zhang, Dahai Yu, Honggui Liu, Ziwei Zhang

**Affiliations:** ^1^College of Veterinary Medicine, Northeast Agricultural University, Harbin 150030, China; ^2^College of Animal Science and Technology, Northeast Agricultural University, Harbin 150030, China; ^3^Key Laboratory of the Provincial Education Department of Heilongjiang for Common Animal Disease Prevention and Treatment, College of Veterinary Medicine, Northeast Agricultural University, Harbin 150030, China

## Abstract

Insulin-like growth factor 1 (IGF1) is a multifunctional cellular regulatory factor that can regulate cell growth and development by mediating growth hormone stimulation. However, the mechanism of IGF1 dysfunction in cardiomyocyte development is seldom reported. To study this, we employed the models of IGF1 knockdown in chicken embryo in vivo and in cardiomyocytes in vitro. We detected the antioxidant capacity, PI3K/Akt pathway, energy metabolism-related genes, and myocardial development-related genes. Our results revealed that the low expression of IGF1 can significantly suppress the antioxidant capacity and increase the ROS (*P* < 0.05) levels, activating the AMPK and PI3K pathway by inhibiting the expression of IRS1. We also found that myocardial energy metabolism is blocked through IGF1, GLUT, and IGFBP inhibition, further inducing myocardial developmental disorder by inhibiting Mesp1, GATA, Nkx2.5, and MyoD expression. Altogether, we conclude that low IGF1 expression can hinder myocardial development through the dysfunction of energy metabolism caused by ROS-dependent FOXO activation.

## 1. Introduction

Insulin-like growth factors (IGFs) are a group of polypeptides with growth-promoting function. The secretory cells are widely distributed in tissues such as the liver, kidney, lung, heart, brain, and intestine [1]. IGFs play an important role in cell proliferation, differentiation, individual growth, and development [2]. The IGF family has two subtypes: insulin-like growth factor 1 (IGF1) and insulin-like growth factor 2 (IGF2). The production of IGF1 is dependent on the growth hormone (GH), which is an important growth factor in life processes. Myocardial development is a complex process that is regulated by complex molecular networks composed of many development-related factors. Many studies have shown that various signal pathways are involved in the development of vertebrate hearts, including the bone morphogenetic protein (BMP), Wnt, Notch, and fibroblast growth factor 4 (FGF 4) signal transduction pathways. The BMP and Wnt signaling pathways play an important role in the development of early mesoderm cells into cardiomyocytes; they act on the cardiac-specific transcription factor GATA4 and Nkx2.5 through a signal cascade process, promoting the differentiation of cardiac precursor cells into cardiomyocytes [3, 4]. Musarò et al. demonstrated that localized synthesis of IGF1 is closely related to skeletal muscle hypertrophy, the molecular pathways of which are similar to those responsible for cardiac hypertrophy [5].

Insulin is a hormone secreted by islet *β* cells, and it is the only hormone that reduces blood sugar and promotes the synthesis of glycogen, fat, and protein in animals [6]. Insulin has been proven to regulate metabolism and growth in the body [7]. The insulin receptor (IR) is a tetramer formed by two alpha subunits and two beta subunits linked by disulfide bonds. The two alpha subunits are located on the outer side of the plasma membrane and have a binding site for insulin; the two beta subunits are transmembrane proteins that play a role in signal transduction. The IR family contains IR, insulin-like growth factor receptor (IGFR), and insulin receptor-related receptor (IRR). Intracellular signaling is initiated by activating intracellular tyrosine kinases through a series of structural conformational changes after IR binding to ligands, which exerts important physiological functions in the body [8]. The cardiac cell membrane is rich in IR, making cardiomyocytes a very important target organ for insulin action. Insulin plays a key role in the regulation of various aspects of cardiovascular metabolism through glucose metabolism, protein synthesis, and vascular tone. The IGF family can regulate cardiac lineage induction by expanding the mesodermal cell population [9]. Bisping et al. demonstrated that although IGF1 is unnecessary for cardiac structure and function, GATA4 must be activated by the IGF1 pathway to exert its function [10].

Conformational changes occur in the beta receptor subunit when insulin binds to IR to form a complex, and this leads to autophosphorylation and activation of tyrosine kinase (TK). The complex phosphorylates insulin receptor substrate (IRS) and activates the phosphatidylinositol 3-kinase (PI3K) pathway and mitogen-activated protein kinase (MAPK) pathway. Insulin augments cardiomyocyte contraction, increases ribosomal biogenesis and protein synthesis, stimulates vascular endothelial growth factor (VEGF), and thereby suppresses apoptosis, promoting cell survival and increasing blood perfusion of the myocardium principally through the PKB/Akt signaling pathway [11]. IGF1 can regulate the process of membrane assembly at the axonal growth cone by activating the PI3K pathway [12]. Zhu et al. found that IGF1 can upregulate VEGF-C in breast cancer by mediating the PI3K/Akt and MAPK/ERK1/2 signaling pathways [13]. Treating the smooth muscle cells of the saphenous vein with IGF1 can induce phosphorylation of PI3K-Akt/PKB and promote proliferation of saphenous vein smooth muscle cells [14].

Organisms can produce free radicals during normal metabolism, and excessive oxygen free radicals can cause damage to human tissues and cellular structures [15, 16]. The free radical balances can be maintained depending on the antioxidant system. The body can mediate the accumulation of excess reactive oxygen species (ROS) through some cell signal transduction, which enhances the expression of many protective proteins in the cell. IGF can sense the changes in ROS levels and thus affect the insulin pathway [17]. Papaiahgari et al. demonstrated that ROS can mediate the activation of nuclear factor erythroid 2-related factor 2 (Nrf2) through the PI3K/Akt pathway [18].

In our previous study, we demonstrated that selenium deficiency disrupted insulin responsiveness through inhibition of the PI3K/Akt pathway by producing excessive oxygen free radicals [19, 20]; meanwhile, selenium deficiency can downregulate the expression of IGF1. However, the role of IGF1 in myocardial development is still less reported; in our present study, we developed models for IGF1 knockdown in cardiomyocyte cultures (siRNA) in vitro and IGF1 knockdown in a chicken embryo model in vivo to detect the effect of IGF1 suppression on energy metabolism, insulin pathways, and myocardial development.

## 2. Materials and Methods

All procedures used in this study were approved by the Institutional Animal Care and Use Committee of Northeast Agricultural University (SRM-11).

### 2.1. Primary Cardiomyocyte Culture

Twelve-day-old chicken embryos were used to obtain primary cardiomyocytes for culture. Subsequent to surface disinfection (using 75% alcohol), the chest was dissected to collect the apical portion of the pericardium (approximately 1/3 of the heart), which was immediately transferred to phosphate-buffered solution (PBS) (4°C) and washed to remove fat, connective tissue, and blood clots. Subsequently, the myocardial tissue was cut into small pieces and washed 3 times with PBS. After enzymatic digestion with collagenase-II (0.1%) for 15 minutes on a constant temperature magnetic stirrer (37°C, 100 r/min) for acclimatization and centrifugation, an equal volume of Dulbecco's Modified Eagle's Medium (DMEM)/F12w containing 10% fetal bovine serum and 1x mycillin was added to terminate digestion. The pellet was digested until the small tissue fragments were completely digested. All supernatants were collected with 300 mesh and 500 mesh filters. The cell suspension was centrifuged at 600 rpm for 5 minutes and resuspended in DMEM/F12w twice in disposable Petri dishes for differential adhesion (the first was 1 h; the second was 1.5 h). Nonadherent cells (cardiomyocytes) were collected, centrifuged at 600 rpm, counted, plated in 6-well plates at 2 × 10^5^, and incubated at 37°C, 5% CO_2_, in an adherent culture incubator for 48 h [21].

### 2.2. Establishment of the IGF1 Knockdown Model In Vitro

Cardiomyocytes attain 80% confluence after approximately 48 h of incubation. In chicken cardiomyocyte primary cultures, which are the same as described in our previous experimental method, IGF1 was knocked down using siRNA (sense 5′-GTTCGTATGTGGAGACAGA-3′, anti-sense 5′-TCTGTCTCCACATACGAAC-3′) subsequent to two washes with Opti-MEM (prewarmed). All cells were randomly divided into two groups: C (control group) and KD (knockdown group). For each group, 3 replicates were prepared with 6 × 10^5^ cardiomyocytes per replicate (*n* = 3). Cells in the knockdown group were transfected with 3 *μ*L of 20 *μ*M siRNA and 3 *μ*L of Lipofectamine RNAi MAX Reagent (Invitrogen) in 2 mL of Opti-MEM. Cells in the control group were treated with 2 mL of Opti-MEM (Invitrogen), which only contain the same volume of Lipofectamine RNAi MAX Reagent. Approximately 48 h posttransfection, the cells were harvested for analysis.

### 2.3. Establishment of the IGF1 Silence Model In Vivo

First, 50 *μ*g of nucleic acid was diluted with pure water without endotoxin to a concentration of 1 *μ*g/*μ*L; the final concentration of glucose was 5%, and the final volume was 100 *μ*L. Then, 25 *μ*L of Entranster™ in vivo reagent was diluted with 50 *μ*L of 10% glucose solution and supplemented with pure water. The final concentration of running glucose was 5%, and the final volume was 100 *μ*L of liquid.

The diluted transfection reagent was added to the diluted nucleic acid solution to form a transfection complex, which was then left at room temperature for 15 min. Ninety hatching eggs were randomly divided into two groups (45 per group), viz., the normal group (N) and siRNA group (Si). The subgerminal cavity of each egg was injected with 1 *μ*g siRNA and sealed with a sealing film. All of the eggs were incubated in a constant temperature incubator. The hearts were taken at 6, 8, and 10 days for subsequent experiments.

### 2.4. Detection of Intracellular ROS Accumulation

Posttransfection, ROS activities were measured using an ROS assay kit (Nanjing Jiancheng Bioengineering Institute, China). First, 10 *μ*M DCFH-DA (2,7-dichlorofurescin diacetate) was added to the culture medium, containing the cell samples to be tested, which was then incubated at a constant temperature (37°C) for 45 min. Then, the medium was discarded, and PBS (37°C preheat) was used to wash the cells three times. Finally, the cells were collected for detecting the activities of ROS at the excitation wavelength of 500 ± 15 nm and emission wavelength of 530 ± 20 nm. The cardiomyocytes were visualized using fluorescence microscopy.

### 2.5. Determination of Oxidative Stress Markers

Cells were grown on 6-well plates at a density of 3 × 10^5^ mL^−1^, collected with jets of saline, and centrifuged at 700 × g; then, the supernatant was collected. The hearts of chicken embryos were taken for homogenization in saline solution and centrifuged at 700 g for 20 min, and the supernatants were collected. The hydrogen peroxide (H_2_O_2_), glutathione (GSH), glutathione peroxidase (GSH-Px), catalase (CAT), malondialdehyde (MDA), induced nitric oxide synthase (iNOS), superoxide dismutase (SOD), and total antioxidant capability (T-AOC) contents were measured by detection kits (Nanjing Jiancheng Bioengineering Institute, Nanjing, China), according to the manufacturer's protocols. The SOD activity was measured at 25°C using autooxidation of pyrogallol in 50 Mm Tris/HCl, pH 8, with 100 mM pyrogallol (Nanjing Jiancheng Bioengineering Institute, Nanjing, China).

### 2.6. Determination of the mRNA Expression of the Genes Related to IGF1, the PI3K/Akt Pathway, Insulin, and Cardiac Differentiation

Total RNA was isolated from heart tissues of three points in time and cardiomyocytes by using the TRIzol reagent according to the manufacturer's instructions (Roche, Basel, Switzerland). The dried RNA pellets were resuspended in 50 *μ*L of diethyl pyrocarbonate-treated water. The concentration and purity of the total RNA were determined using a spectrophotometer. cDNA was synthesized from 5 *μ*g of the total RNA using oligo-dT primers and Superscript II reverse transcriptase according to the manufacturer's instructions (Promega, Beijing, China). cDNA was diluted at a ratio of 1 : 5 with sterile water and stored at −80°C.

Primer Premier Software (PREMIER Biosoft International, USA) was used to design specific primers for IGF1 and AMP-activated protein kinase (AMPK), phosphatidylinositol 3-kinase (PI3K), c-Jun N-terminal kinase (JNK), threonine-protein kinase (Akt), forkhead box protein (FOXO), insulin-like growth factor 1 receptor (IGF1R), glucose transporter-1 (GLUT1), glucose transporter-3 (GLUT3), glucose transporter-8 (GLUT8), insulin-like growth factor-binding protein-1 (IGFBP1), insulin-like growth factor-binding protein-2 (IGFBP2), insulin-like growth factor-binding protein-3 (IGFBP3), insulin-like growth factor-binding protein-4 (IGFBP4), insulin-like growth factor-binding protein-5 (IGFBP5), insulin-like growth factor-binding protein-7 (IGFBP7), insulin receptor (IR), insulin receptor substrate-1 (IRS1), MyoD, myogenin (MyoG), cardiac transcription factor mesoderm posterior 1 (Mesp1), myogenic factor-5 (MYF5), myogenic factor-6 (MYF6), GATA-binding protein 4 (GATA4), GATA-binding protein 6 (GATA6), NK2 homeobox 5 (Nkx2.5), and glyceraldehyde-3-phosphate dehydrogenase (GAPDH) based on known chicken sequences (Table 1). First, general PCR was performed to confirm the specificity of the primers. Quantitative real-time PCR (qPCR) was then performed with a Roche detection system (Applied Biosystems, Foster City, CA). The reactions were conducted in a 20 *μ*L reaction mixture containing 10 *μ*L of 2x SYBR Green I PCR Master Mix (Roche, Basel, Switzerland), 2 *μ*L of cDNA, 0.4 *μ*L of each primer (10 *μ*M), 0.4 *μ*L of 50x ROX reference Dye II, and 6.8 *μ*L of PCR-grade water. The PCR procedure for IGF1 and AMPK, PI3K, JNK, Akt, FOXO, IGF1R, GLUT1, GLUT3, GLUT8, IGFBP1, IGFBP2, IGFBP3, IGFBP4, IGFBP5, IGFBP7, IR, IRS1, MyoD, MyoG, Mesp1, MYF5, MYF6, GATA4, GATA6, Nkx2.5, and GAPDH consisted of 95°C for 30 s followed by 40 cycles of 95°C for 15 s, 60°C for 30 s, and 60°C for 30 s. For each PCR, Dissociation Curve 1.0 Software (Applied Biosystems) was used to analyze the dissociation curves in order to detect and eliminate possible primer dimers and nonspecific amplification.

### 2.7. Determination of the Protein Expression of the Proteins Related to IGF1, the PI3K/Akt Pathway, Insulin, and Cardiac Differentiation

For total protein extraction, protein lysates were subjected to 15% SDS-polyacrylamide gel electrophoresis under reducing conditions. The separated proteins were then transferred to a nitrocellulose membrane for 2 h at 100 mA in a transfer apparatus containing Tris-glycine buffer and 20% methanol. The membrane was blocked with 5% skim milk for 24 h and incubated overnight with diluted primary antibodies against IGF1 (1 : 500, Proteintech, China), PI3K (1 : 1000, Santa Cruz Biotechnology, USA), Akt (1 : 500, Santa Cruz Biotechnology, USA), P-Akt (1 : 500, Proteintech, China), FOXO (1 : 1000, Santa Cruz Biotechnology, USA), P-FOXO (1 : 1000, Santa Cruz Biotechnology, USA), JNK (1 : 1000, Santa Cruz Biotechnology, USA), P-JNK (1 : 1000, Santa Cruz Biotechnology, USA), 14-3-3 (1 : 1000, Abcam, Cambridge, UK), P-14-3-3 (1 : 1000, Santa Cruz Biotechnology, USA), GLUT3 (1 : 300, Santa Cruz Biotechnology, USA), IGF1R*β* (1 : 500, Proteintech, China), IGFBP2 (1 : 500, Proteintech, China), MyoG (1 : 1000, Abcam, Cambridge, UK), and MyoD (1 : 1000, Abcam, Cambridge, UK) followed by a horseradish peroxidase- (HRP-) conjugated secondary antibody against rabbit (IGF1, PI3K, Akt, P-Akt, FOXO, P-FOXO, JNK, P-JNK, 14-3-3, P-14-3-3, GLUT, IGF1R*β*, IGFBP2, MyoG, and MyoD) IgG (1 : 5000, Santa Cruz Biotechnology, USA). To verify equal loading of the samples, the membrane was incubated with a monoclonal GAPDH antibody (1 : 1500, Santa Cruz Biotechnology, USA), followed by an HRP-conjugated goat anti-mouse IgG (1 : 3000) secondary antibody. The signal was detected with X-ray films (TransGen Biotech Co., Beijing, China). The optical density (OD) of each band was determined using an Image VCD gel imaging system, and the relative abundance of IGF1, PI3K, Akt, P-Akt, FOXO, P-FOXO, JNK, P-JNK, 14-3-3, P-14-3-3, GLUT3, IGF1R*β*, IGFBP2, MyoG, and MyoD proteins was calculated and presented as the ratios of OD of each of these proteins to that of GAPDH.

### 2.8. Measurement of ATP

Cardiomyocytes were grown on 6-well plates at a density of 2 × 10^5^ cells/mL, gathered with the lysis solution, and centrifuged at 700 × g. The supernatant was collected, resuspended by salt water and incubated for 35 min at 25°C. The level of adenosine triphosphate (ATP) in the cardiomyocytes was measured by using an ATP detection kit (Nanjing Jiancheng Bioengineering Institute, Nanjing, China) according to the manufacturer's instructions. The detection was carried out using an ultraviolet spectrophotometer (Synergy NEO, BioTek Instruments) with a detection wavelength of 636 nm.

### 2.9. Histopathological Examination

Cardiomyocytes were grown at a density of 2 × 10^5^ cells/mL and then washed with PBS three times; 4% paraformaldehyde solution was added in a 24-well plate for cell fixing. After 12 h, the 4% paraformaldehyde solution in the 24-well plate was removed and 0.01 M PBS was added; then, the wells were soaked for 5 min × 3 times. Hematoxylin staining solution was added to the wells and immersed for 1 min. The staining solution was removed, and distilled water was added to soak for 5 min. The distilled water was then removed and placed in 1% hydrochloric acid alcohol. After 1-3 s, it was aspirated. Tap water was added to soak for 5 min to return the cells to blue. Then, the tap water was removed, and Yihong dye solution was added to soak for 1 min. The eosin staining solution was aspirated and soaked in distilled water for 1 min. The climbing pieces were removed, and glycerol ethanol was added dropwise. The staining effect was observed under a microscope and photographed under a 200x microscope [22].

The myocardial tissues were rapidly fixed in 10% formaldehyde for at least 24 h and embedded in paraffin for microscopic examination. From the prepared paraffin blocks, sections (5 *μ*m thick) were cut, obtained, and stained with hematoxylin and eosin (H.E.) for light microscopic observation.

### 2.10. High-Resolution Respirometry of Mitochondrial Function

Cardiomyocytes were grown on 6-well plates at a density of 2 × 10^5^ cells/mL. All cells were randomly divided into two groups: C (control group) and KD (knockdown group). The cells in the knockdown group were transfected with 3 *μ*L of 20 *μ*M siRNA and 3 *μ*L of Lipofectamine RNAi MAX Reagent (Invitrogen) in 2 mL of Opti-MEM. The cells in the control group were treated with 2 mL of Opti-MEM (Invitrogen) containing the same volume of Lipofectamine RNAi MAX Reagent. Approximately 48 h posttransfection, the cells were harvested for analysis, gathered with the lysis solution, and centrifuged at 700 × g. The supernatant was collected and resuspended by the medium for high-resolution respirometry. The mitochondrial respiratory function was analyzed in a two-channel titration injection respirometer (Oxygraph-2k; Oroboros Instruments, Innsbruck, Austria). The cell suspension was transferred separately to oxygraph chambers at a final density of approximately 2×10^5^ cells/mL. After a short stabilization period, the chambers were closed and data were recorded using DatLab software 5.2 (Oroboros Instruments, Innsbruck, Austria).

### 2.11. Statistical Analysis

Statistical analyses were performed using GraphPad Prism 5.0 software, and all data was assessed using the unpaired *t*-test, where *P* < 0.05 was considered a statistically significant difference.

## 3. Results

### 3.1. Development of an IGF1 Knockdown Model in Cells and Chicken Embryos

**Figure 1:**
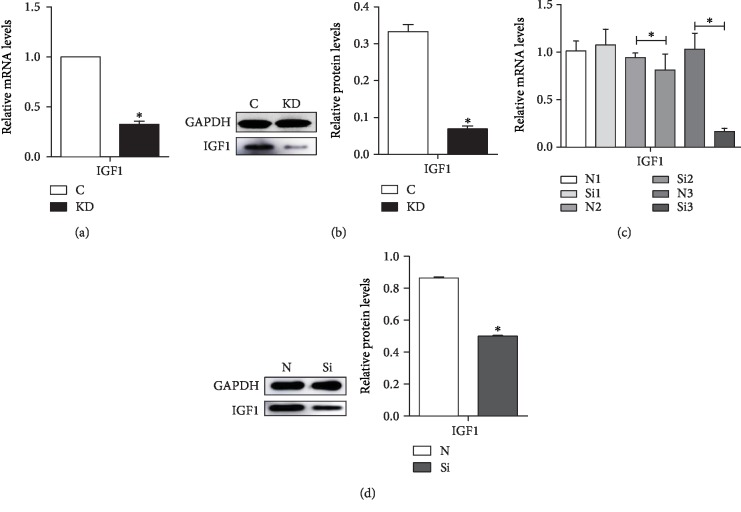
The effects of IGF1 knockdown on the mRNA levels (a) and protein levels (b) of the IGF1 gene in cardiomyocytes and the effects of IGF1 silencing on the mRNA levels at 6, 8, and 10 days (c) and on the protein levels (d) of the IGF1 gene in the myocardium. The results were calculated from at least three independent experiments, *n* = 3. The data are expressed as the means ± SD. C indicates the control group; KD indicates the knockdown group in vitro; N indicates the normal group; Si indicates the knockdown group in vivo. ∗ indicates a significant difference from the corresponding control (*P* < 0.05).

The mRNA and protein levels of IGF1 were significantly decreased (*P* < 0.05) in the KD group (Figures 1(a) and 1(b)). The results confirmed that we successfully established the model of IGF1 knockdown in vitro.

The mRNA levels of IGF1 were detected at 6, 8, and 10 days, and we found that the expression of IGF1 was significantly decreased at 10 days. For further verification, we took chicken embryos at 10 days to detect the protein level of IGF1. Compared with the N group, the mRNA levels in the Si group decreased (*P* < 0.05) at 8 days and 10 days (Figure 1(c)). The Si group exhibited significantly decreased protein levels compared with the N group (Figure 1(d)). The results confirmed that we successfully established the IGF1 silence model in vivo.

### 3.2. Detection of the Antioxidant Capacity in Cells and Chicken Embryos

**Figure 2:**
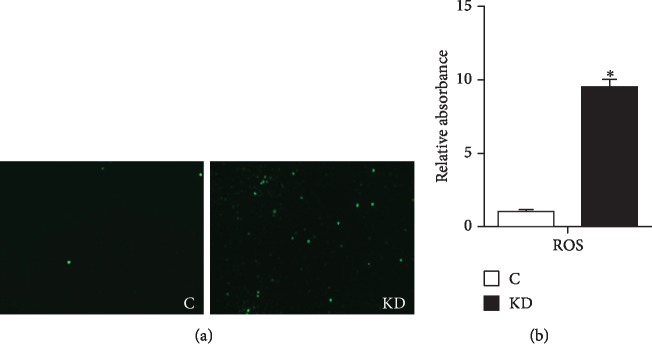
(a) ROS generation was performed by immunofluorescence using DCFH-DA (green fluorescence, 5 mM) in cells. C indicates the control group; KD indicates the knockdown group. Cardiomyocytes were visualized using fluorescence microscopy. (b) The effects of IGF1 knockdown on the ROS levels in cardiomyocytes were detected by using a fluorescence microplate reader. C indicates the control group; KD indicates the knockdown group. ∗ shows a significant difference from the corresponding control (*P* < 0.05). *n* = 3.

**Figure 3:**
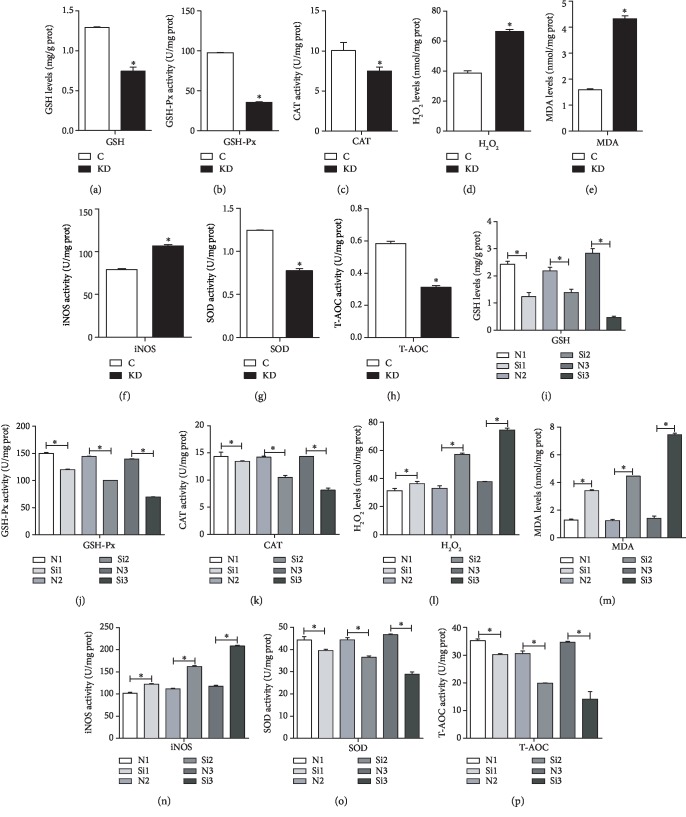
(a–h) Oxidative stress markers of the GSH, GSH-Px, CAT, H_2_O_2_, MDA, iNOS, SOD, and T-AOC contents were measured in cardiomyocytes. (i–p) Oxidative stress markers of GSH, GSH-Px, CAT, H_2_O_2_, MDA, iNOS, SOD, and T-AOC contents were measured in the myocardium. C indicates the control group; KD indicates the knockdown group in vitro; N indicates the normal group; Si indicates the knockdown group in vivo. ∗ shows a significant difference from the corresponding control (*P* < 0.05). *n* = 3.

To assess the relationship between oxidative stress and IGF1, the production of ROS, the levels of H_2_O_2_, MDA, and T-AOC and the activities of GSH, GSH-Px, SOD, CAT, and iNOS were measured in cardiomyocytes. As presented in Figure 2, the ROS activities were significantly increased (*P* < 0.05) compared with the C group. The levels of H_2_O_2_, MDA, and iNOS were significantly increased in the KD group (*P* < 0.05) (Figures 3(d)–3(f)). The levels of GSH, GSH-Px, CAT, SOD, and T-AOC in the KD group were significantly lower than those in the C group (*P* < 0.05) (Figures 3(a)–3(c), 3(g), and 3(h)).

To further demonstrate the antioxidant capacity of IGF1 silence in vivo, we also studied the antioxidant capacity of the myocardium. As shown in Figure 3, the levels of H_2_O_2_, MDA, and iNOS were significantly increased in the Si group (*P* < 0.05) at 6, 8, and 10 days (Figures 3(l)–3(n)). The levels of GSH, GSH-Px, CAT, SOD, and T-AOC in the Si group were significantly lower than those in the N group (*P* < 0.05) at 6, 8, and 10 days (Figures 3(i), 3(j), 3(l), 3(o), and 3(p)).

### 3.3. Protein and mRNA Expression of the PI3K/Akt Pathway-Related Genes in Cells and Chicken Embryos

**Figure 4:**
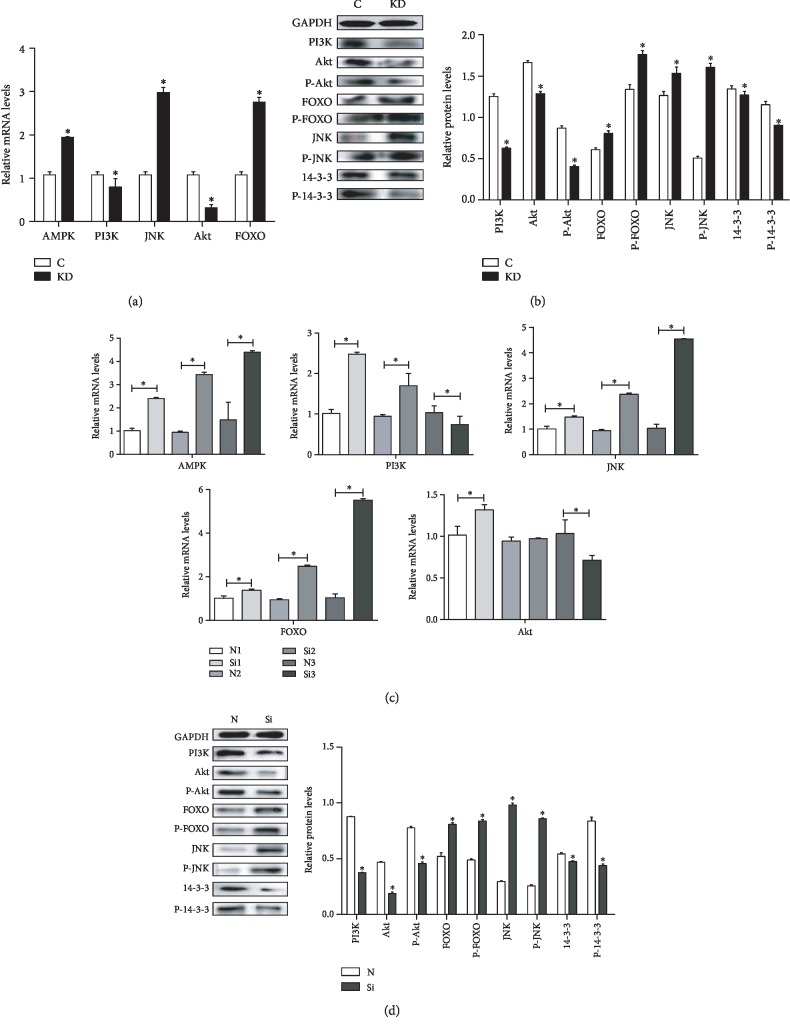
The effects of IGF1 knockdown on the mRNA levels (a) and protein levels (b) of PI3K-related genes in cardiomyocytes and the effects of IGF1 silencing on the mRNA levels at 6, 8, and 10 days (c) and on the protein levels (d) of PI3K-related genes in the myocardium. C indicates the control group; KD indicates the knockdown group in vitro; N indicates the normal group; Si indicates the knockdown group in vivo. GAPDH was selected as the reference. ∗ shows a significant difference from the corresponding control (*P* < 0.05). *n* = 3.

To examine whether IGF1 knockdown changed the expressions of the PI3K/Akt pathway-related genes, we detected the mRNA expression levels of AMPK, PI3K, JNK, Akt, and FOXO as well as the protein expression levels of FOXO, P-FOXO, JNK, P-JNK, PI3K, Akt, P-Akt, 14-3-3, and P-14-3-3. The effects of IGF1 knockdown on the mRNA abundance of PI3K-related genes in chicken cardiomyocytes are shown in Figure 4(a). Compared with the C group, the qPCR results revealed that the mRNA expression of AMPK, JNK, and FOXO was significantly increased (*P* < 0.05) in the KD group. However, the mRNA expression of PI3K and Akt was decreased (*P* < 0.05). The results revealed that compared with the C group, the protein expression of FOXO, P-FOXO, JNK, and P-JNK in the KD group was significantly increased (*P* < 0.05). However, the protein expression of PI3K, Akt, P-Akt, 14-3-3, and P-14-3-3 decreased in the KD group (*P* < 0.05) (Figure 4(b)).

Moreover, we detected the effects of IGF1 silencing on the mRNA abundance of PI3K-related genes (AMPK, PI3K, JNK, Akt, and FOXO) in the myocardium of chicken embryos as shown in Figure 4(c). The qPCR results revealed that the mRNA expression of AMPK, JNK, and FOXO was significantly increased (*P* < 0.05) in the Si group at 6, 8, and 10 days. The mRNA expression of PI3K increased at 6 and 8 days but significantly decreased (*P* < 0.05) at 10 days. The mRNA expression of Akt increased at 6 days, showed no significant change at 8 days, and significantly decreased (*P* < 0.05) at 10 days. A western blot analysis was performed to determine the protein expression of PI3K-related genes. The results revealed that compared with the N group, the protein expression of FOXO, P-FOXO, JNK, and P-JNK in the Si group was significantly increased (*P* < 0.05). However, the protein expression of PI3K, Akt, P-Akt, 14-3-3, and P-14-3-3 decreased in the Si group (*P* < 0.05) (Figure 4(d)).

### 3.4. Protein and mRNA Expression of Insulin-Related Genes in Cells and Chicken Embryos

**Figure 5:**
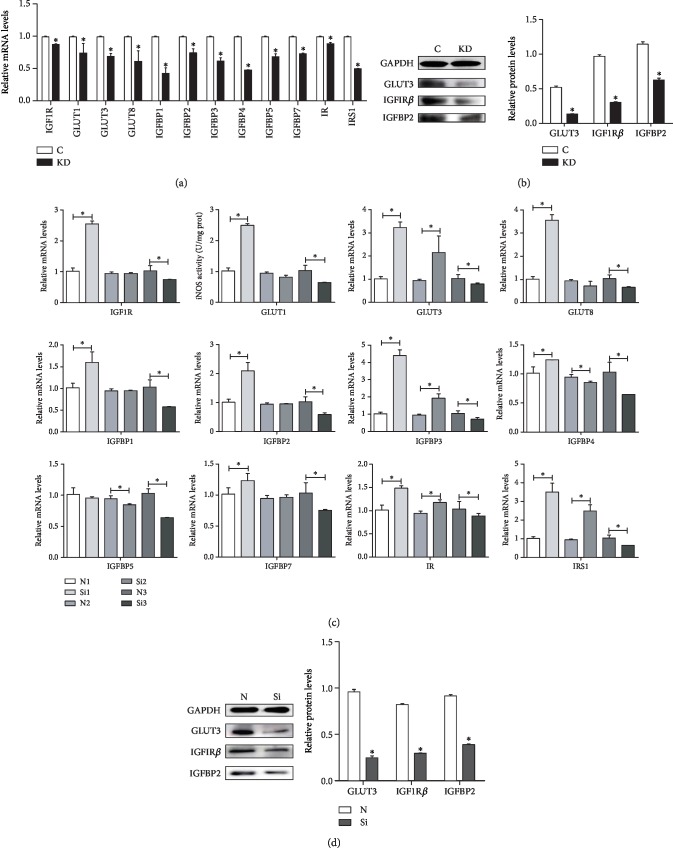
The effects of IGF1 knockdown on the mRNA levels (a) and protein levels (b) of insulin-related genes in cardiomyocytes and the effects of IGF1 silencing on the mRNA levels at 6, 8, and 10 days (c) and on the protein levels (d) of insulin-related genes in the myocardium. C indicates the control group; KD indicates the knockdown group in vitro; N indicates the normal group; Si indicates the knockdown group in vivo. GAPDH was selected as the reference. ∗ shows a significant difference from the corresponding control (*P* < 0.05). *n* = 3.

We also examined the effects of IGF1 knockdown on the mRNA abundance of insulin-related genes (IGF1R, GLUT1, GLUT3, GLUT8, IGFBP1, IGFBP2, IGFBP3, IGFBP4, IGFBP5, IGFBP7, IR, and IRS1) in chicken cardiomyocytes (Figure 5(a)); the qPCR results revealed that the mRNA expression of IGF1R, GLUT1, GLUT3, GLUT8, IGFBP1, IGFBP2, IGFBP3, IGFBP4, IGFBP5, IGFBP7, IR, and IRS1 was significantly decreased (*P* < 0.05) in the KD group. A western blot analysis was performed to determine the protein expression of insulin-related genes. The results revealed that compared with the C group, the protein expression of GLUT3, IGF1R*β*, and IGFBP2 in the KD group was significantly decreased (*P* < 0.05) (Figure 5(b)).

Furthermore, we examined the effects of IGF1 knockdown on the mRNA abundance of insulin-related genes (IGF1R, GLUT1, GLUT3, GLUT8, IGFBP1, IGFBP2, IGFBP3, IGFBP4, IGFBP5, IGFBP7, IR, and IRS1) in the myocardium of chicken embryos as shown in Figure 5(c). The qPCR results revealed that the mRNA expression of IGF1R, GLUT1, GLUT8, IGFBP1, IGFBP2, and IGFBP7 increased in the Si group at 6 days, showed no significant change at 8 days, and significantly decreased (*P* < 0.05) at 10 days. The mRNA expression of GLUT3, IGFBP3, IR, and IRS1 increased at 6 and 8 days, but significantly decreased (*P* < 0.05) at 10 days. The mRNA expression of IGFBP4 increased at 6 days but significantly decreased at 8 and 10 days. The mRNA expression of IGFBP5 significantly decreased (*P* < 0.05) at 8 and 10 days. A western blot analysis was performed to determine the protein expression of insulin-related genes. The results revealed that compared with the N group, the protein expression of GLUT3, IGF1R*β*, and IGFBP2 in the Si group was significantly decreased (*P* < 0.05) **(**Figure 5(d)).

### 3.5. The Oxygen Consumption Rate and ATP Content in Myocardial Cells

**Figure 6:**
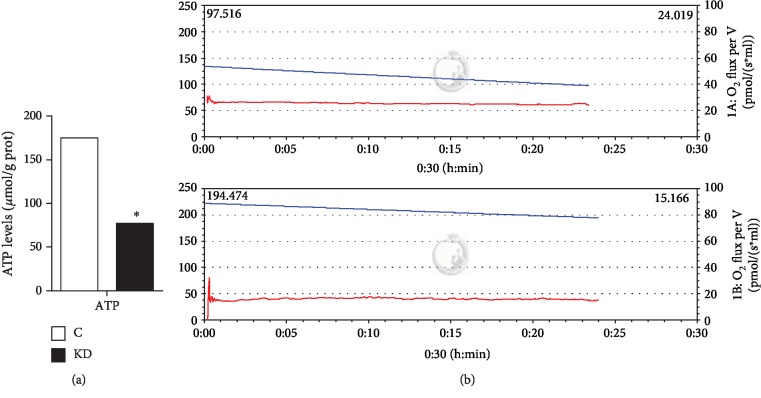
(a) The level of ATP was determined to investigate the function of energy metabolism. The data are represented as the means ± SD. Samples with an asterisk (∗) represent significant differences (*P* < 0.05), *n* = 3. The myocardial oxygen consumption rate results are shown in (b). The red curve indicates the oxygen consumption, and the blue curve indicates the oxygen concentration. C indicates the control group; KD indicates the knockdown group.

To assess the effect of IGF1 knockdown on energy metabolism, the oxygen consumption rate and ATP content were detected. The results revealed that IGF1 knockdown significantly decreased the ATP content (Figure 6(a)). The results of the oxygen consumption rate revealed that the oxygen consumption rate of the IGF1 knockdown group was 15.166 while the oxygen consumption rate of the control group was 24.019, indicating that IGF1 knockdown significantly decreased the myocardial oxygen consumption rate under the same conditions and the same initial oxygen concentration (Figure 6(b)).

The instantaneous oxygen consumption rates of the cardiomyocytes for different groups are provided in Table 2.

### 3.6. Protein and mRNA Expression of Cardiac Differentiation-Related Genes in Cells and Chicken Embryos

**Figure 7:**
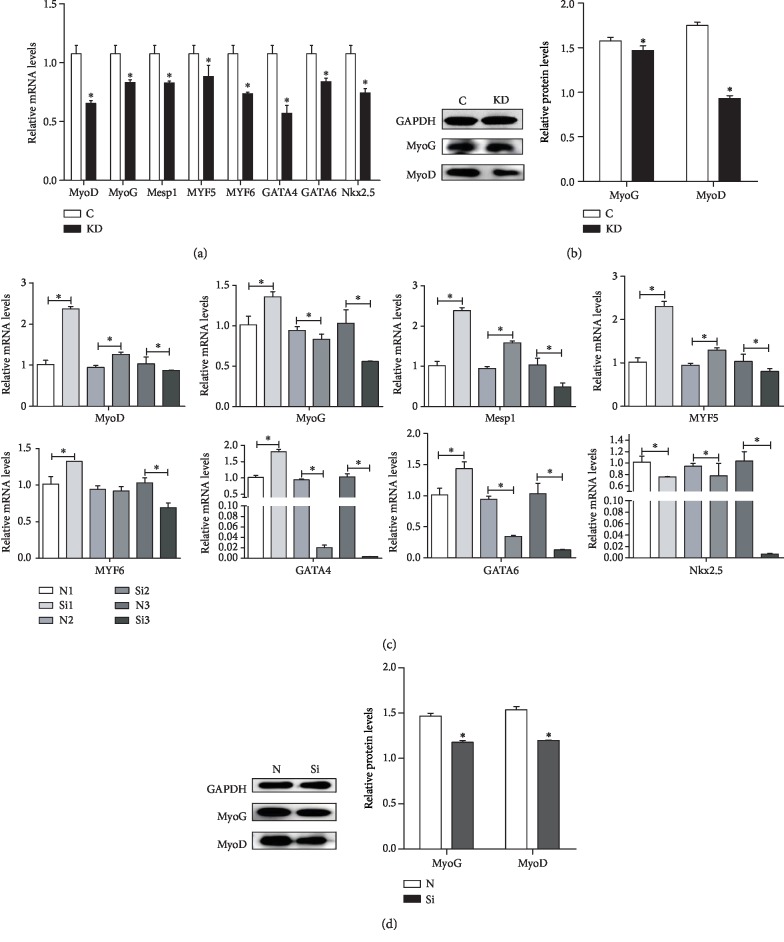
The effects of IGF1 knockdown on the mRNA levels (a) and protein levels (b) of cardiac differentiation-related genes in cardiomyocytes and the effects of IGF1 silencing on the mRNA levels at 6, 8, and 10 days (c) and on the protein levels (d) of cardiac differentiation-related genes in the myocardium. C indicates the control group; KD indicates the knockdown group in vitro; N indicates the normal group; Si indicates the knockdown group in vivo. GAPDH was selected as the reference. ∗ shows a significant difference from the corresponding control (*P* < 0.05). *n* = 3.

The effects of IGF1 knockdown on the mRNA abundance of cardiac differentiation-related genes (MyoD, MyoG, Mesp1, MYF5, MYF6, GATA4, GATA6, and Nkx2.5) in chicken cardiomyocytes are shown (Figure 7(a)), and the qPCR results revealed that the mRNA expression of MyoD, MyoG, Mesp1, MYF5, MYF6, GATA4, GATA6, and Nkx2.5 was significantly decreased (*P* < 0.05) in the KD group. A western blot analysis was performed to determine the protein expression of cardiac differentiation-related genes. The results revealed that compared with the C group, the protein expression of MyoG and MyoD in the KD group was significantly decreased (*P* < 0.05) (Figure 7(b)).

The effects of IGF1 knockdown on the mRNA abundance of cardiac differentiation-related genes (MyoD, MyoG, Mesp1, MYF5, MYF6, GATA4, GATA6, and Nkx2.5) in the myocardium of chicken embryos are shown in Figure 7(c). The qPCR results revealed that the mRNA expression of MyoD, Mesp1, and MYF5 increased in the Si group at 6 and 8 days, but significantly decreased (*P* < 0.05) at 10 days. The mRNA expression of MyoG, GATA4, and GATA6 increased at 6 days, but significantly decreased at 8 and 10 days. The mRNA expression of MYF6 increased at 6 days, showed no significant change at 8 days, and significantly decreased (*P* < 0.05) at 10 days. The mRNA expression of Nkx2.5 decreased (*P* < 0.05) at 6, 8, and 10 days. A western blot analysis was performed to determine the protein expression of cardiac differentiation-related genes. The results revealed that compared with the N group, the protein expression of MyoG and MyoD in the Si group was significantly decreased (*P* < 0.05) (Figure 7(d)).

### 3.7. Intracellular Morphological Observation and H.E. Stain in Cells and Chicken Embryos' Myocardium

**Figure 8:**
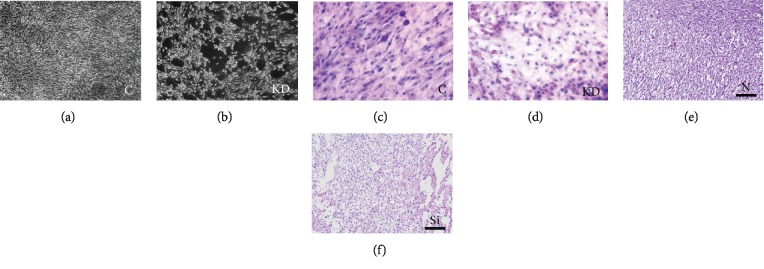
Morphological observation was performed in cardiomyocytes transfected with Opti-MEM (C group, a) and siRNA (KD group, b) for 24 h. Cardiomyocytes were stained by H.E., and the results are shown in (c) and (d). H.E. staining for myocardial tissues in the N group (e) and the Si group (f). C indicates the control group; KD indicates the knockdown group in vitro; N indicates the normal group; Si indicates the knockdown group in vivo.

As observed under a microscope, normal cardiomyocytes were fusiform and tightly connected and the whole looked similar to paving stones accompanied by protruding pseudopodia that stretched out between cells, interweaving into a mesh in the control group (Figure 8(a)). In the KD group, as the density of cell growth decreased, the volume of the cardiomyocytes and intercellular junctions was evidently reduced. We observed that myocardial fibers and muscle fiber bundles were disintegrated, and the pseudopodia between cells did not interweave into a mesh in the KD group (Figure 8(b)). We observed myocardial cells stained by hematoxylin and eosin (H.E.). The cardiomyocytes in the control group displayed normal morphologies (Figure 8(c)). However, many slender cardiomyocytes appeared in the IGF1 knockdown group (Figure 8(d)), which indicated that cardiomyocyte development was blocked.

Myocardial injury and ultrastructural damage are shown in Figure 8(f). Blood vessel rupturing and increased tissue gaps were observed in the IGF1-deficient chicken heart group more than in the normal group (Figure 8(e)).

## 4. Discussion

IGF1 is a polypeptide neurotropic factor with a structure and function similar to insulin. IGF1 is a single-chain protein that promotes cell differentiation and proliferation, and it has a wide range of biological functions and participates in the regulation of various organs. IGF1 plays an important role in the development of human and vertebrate embryos [23]. In the present study, we established an IGF1 knockdown model in vivo and in vitro through transfection of small interfering RNA (siRNA) and the mRNA and protein levels of IGF1 were detected to support this point. Significant damage to myocardial tissue, rupturing of blood vessels, and increased tissue gaps were observed in IGF1-deficient chicken heart through histopathological observation, demonstrating that IGF1 suppression leads to dysplasia of cardiomyocytes and myocardial tissue.

The concentration of free radicals is low under physiological conditions, which is important to cell signal regulation, metabolism, survival, and apoptosis [24, 25]. However, a large number of free radicals are induced when organisms are stimulated by physical factors or exogenous chemical substances; free radicals can covalently bind to biomacromolecules and peroxidize biofilm lipids to produce various toxic effects [26], which can lead to the occurrence of many diseases such as myocardial infarction [27, 28] and various cancers [29]. Oxygen free radicals can make lipid fatty acids into lipid peroxides and further decompose into a series of complex compounds, including MDA; therefore, the level of lipid oxidation can be detected by the level of MDA [30]. iNOS is an oxidative stress (free radical) that utilizes nitric oxide, which can be produced when cells are stimulated and activated. The cells can form a complex antioxidant enzyme defense system, which mainly includes SOD, CAT, GSH, and so on, to protect the body from peroxidative damage [26, 31]. SOD can eliminate free radicals in the body and protect cells from free radical damage. The level of SOD activity reflects the ability of antifree radicals. SOD can convert superoxide anion (O2^−^) into H_2_O_2_, and CAT converts H_2_O_2_ into water such that toxic O_2_^−^ and H_2_O_2_ are converted into harmless water molecules [32, 33]. GSH is catalyzed to convert to oxidized glutathione (GSSH) with the assistance of GSH-Px, which can reduce oxidized substances and relieve their toxicity [34, 35]. In recent years, the antioxidant functions of IGF1 have been gradually discovered; Tumati et al. found that low IGF1 can induced excessive ROS, which will be further moderated by JNK-induced epithelial cytoprotection [36]. ROS, as an important endogenous stimulator in the body, can stimulate multiple pathways including myocardial development. Huk et al. found that ROS serves as secondary messengers to influence cardiac valve development [37]. ROS may be involved in adverse cardiac remodeling [38]. In our present study, we found that decreasing expression of IGF1 results in increasing ROS generation, suggesting that IGF1 may be involved in the regulation of ROS and the occurrence of oxidative stress. IGF1 significantly decreased the CAT, SOD, GSH, and GSH-Px activities in vivo and in vitro. These changes were accompanied by reduced T-AOC, which is an important indicator for determining the body's antioxidant capacity; meanwhile, the expression of iNOS was significantly increased. All of these results demonstrate that IGF1 suppression can significantly reduce the body's antioxidant capacity and enrich many oxygen free radicals in the body; this may be one of the important causes of myocardial damage and dysplasia.

FOXO, a highly conserved transcriptional regulatory protein, is a downstream protein of the PI3K/Akt and AMPK/14-3-3 signaling pathways [39, 40]. The PI3K/Akt/FOXO signaling pathway is involved in the regulation of various physiological processes such as proliferation, apoptosis, and insulin resistance. The PI3K/Akt pathway can be activated by several of these stimuli, and ROS is one of the most important factors. Adipogenic differentiation can be mediated through activation of the PI3K/Akt pathway by oxidative stress in primary rat osteoblasts [41]. Stitt et al. found that the IGF1/PI3K/Akt pathway plays an important role in preventing the expression of ubiquitin ligases by inhibiting FOXO transcription factors [42]. AMPK is an important protein kinase in eukaryotic cells. The energy regulation of AMPK can maintain normal ATP levels in cardiomyocytes [43]. AMPK regulates the utilization of energy throughout the body, and it is considered the energy regulator [44]. FOXO is an important downstream molecule of AMPK involved in the signal transduction and regulation of various cellular biological processes such as inhibiting cell proliferation and promoting apoptosis [45, 46]. IGF1 stimulation can decrease the level of AMPK phosphorylation in F1 and F3/4 granulosa cells [47]. Hinchy proved that the ROS released from mitochondria can activate AMPK indirectly [48]. Furthermore, FOXO has been proven to be required in endothelial but not myocardial cell lineages during cardiovascular development [49]. Evans-Anderson et al. demonstrated that a FOXO transcription factor is a negative regulator of cardiomyocyte proliferation during heart development [50]. In our present study, we found that IGF1 depletion inhibited the expression of IRS1, PI3K, and Akt and upregulated both the mRNA and protein expression of AMPK and FOXO; meanwhile, the expression of JNK significantly increased in the IGF1 suppression group, which is important upstream of IRS1. All of these results suggest that IGF1 knockdown can activate FOXO through inhibiting the expression of IRS1. Combined with the results of ROS, we concluded that IGF1 suppression triggers ROS release to activate FOXO, which further inhibits myocardial development.

Mitochondria, as an energy converter in animal cells, are a main site for intracellular oxidative phosphorylation and ATP formation. Pawlikowska et al. showed that mitochondria are essential organelles for insulin-mediated muscle formation and that insulin stimulates mitochondria and promotes mitochondrial function [51]. Insulin-like growth factor-binding proteins (IGFBPs) are important parts of the IGF family; IGFBPs cannot bind with insulin but rather form a complex with IGFs. They can regulate the normal growth of embryonic and postnatal organs, which is also a very important tool for IGF1 transport and storage. The IGF1-IGFBP complex will decompose and release IGF1, which will further combine with IGF1R in the cell membrane under the catalysis of IR [52]. IGF1 is protected from decomposition by its high affinity with IGFBP, prolonging the half-life of IGF1 in the body's circulation cells. IGFBPs avoid the body's negative effects for insulin overdose by reducing the concentration of free IGF1 in the blood. In addition, studies have shown that IGFBP can help IGF1 recognize target cell and regulate the activity of IGF1 [53]. The IGF1-IGFBP complex can cause a cascade of various phosphorylation processes in the cell, ultimately leading to the entry of the glucose transporter molecules (GLUT1, 3, and 8) into the cell membrane and increasing the rate of glucose transport in the cell. Glucose transport is mainly dependent on GLUT, and it plays an important role in regulating glucose transport and maintaining cardiac energy balance [54]. IGF1RS/IRS signaling regulates cellular energy metabolism through the PI3K/Akt signaling pathway, and its downstream genes have been found [55]. FOXO plays an important role in mediating the effects of insulin and growth factors on diverse physiological functions [56]. In the present study, we demonstrated that IGF1 knockdown significantly decreased the expression of GLUT3 and IGFBP, indicating that IGF1 suppression blocks energy metabolism. To further verify these results, we also detected the ATP content and oxygen consumption rate in two different groups; the results revealed that IGF1 knockdown can significantly impede the ability of using oxygen for cardiomyocytes, thereby reducing the production of ATP. Considering our results showing FOXO activation by IGF1 knockdown, we conclude that FOXO can inhibit myocardial development by interfering with myocardial energy metabolism.

Myogenic regulatory factors (MRFs) are a class of muscle-specific regulators that determine the function of cell-directed differentiation. MRFs are essential for muscle development in vertebrates [56]. MRFs include MYF5, MyoD, MyoG, and MRF4; the function of MRFs is transforming mesenchymal stem cells into myoblasts, which will further activate and maintain the differentiation state of myocytes. Each member of the MRF family plays different roles at different states. MyoD and MYF5 play an important role in the early proliferative phase of myocytes [57]. MYF5 is responsible for myoblast proliferation, and MyoD regulates the differentiation process for myoblasts. MyoG and MRF4 can drive terminal differentiation. The proliferation process of myoblasts is normal after knocking out MyoG, but the subsequent differentiation process is significantly inhibited [58]. Mesp1 and Mesp2 are key genes in regulating cardiac differentiation. Mesp1 can directly activate other genes in the cardiac core by binding to other gene promoters in cardiomyocytes. Mesp1 and Mesp2 reduction can impede heart development [59]. GATA4 and Nkx2.5 are transcription factors involved in heart development, and overexpression of GATA4 can accelerate myocardial differentiation [60]. Early embryo development requires the production and expenditure of large amounts of cellular energy for cell growth. Insulin can regulate skeletal muscle cell differentiation by mediating the activation of MAPK and PKB phosphorylation [61]. Montarras et al. demonstrated that insulin or IGF is necessary for cell differentiation [62]. Cellular energy metabolism that contains fatty acid is involved in fetal heart development [63]. In the present study, we found that IGF1 knockdown can significantly decrease the expression of MyoD, Mesp1, MYF5, MYF6, GATA4, GATA6, and Nkx2.5, indicating that IGF1 suppression can block myocardial development. We suggest that reducing energy metabolism inhibits the expression of myocardial development to factors through comprehensive results on energy metabolism.

In summary, we conclude that IGF1 knockdown hinders myocardial development through the energy metabolism dysfunction caused by ROS-dependent FOXO activation in the chicken heart. Our results indicate a novel hypothesis for IGF1 in the development of cardiomyocytes.

## Figures and Tables

**Table 1:** The primers used in the present study.

Target gene	Primer sequence (5′-3′)
IGF1	Forward 5′-GCTTTTGTGATTTCTTGAAGGTGAA-3′
	Reverse 5′-CATACCCTGTAGGCTTACTGAAGTA-3′

AMPK	Forward 5′-CCATCTGTCATTAGTCTTCTG-3′
	Reverse 5′-AGGCTTCGTCATCAATCAT-3′

PI3K	Forward 5′-GTCCTTGAGCCACTGATG-3′
	Reverse 5′-TGTTGCCTTACGGTTGTT-3′

Akt	Forward 5′-AGGAGGAAGAGATGATGGAT-3′
	Reverse 5′-GAATGGATGCCGTGAGTT-3′

JNK	Forward 5′-CAGATAAGCAGTTAGATGAGAG-3′
	Reverse 5′-GACAGATGACGACGAAGAT-3′

FOXO	Forward 5′-CAGCAATGTCAAGGAGAGCA-3′
	Reverse 5′-TGAAGAGGTTGTCCGAGTCC-3′

IGF1R	Forward 5′-GCGTGAGAGGATAGAGTTC-3′
	Reverse 5′-TGTTGGCGTTGAGGTATG-3′

GLUT1	Forward 5′-TAGTACTGGAGCAGGTGGCAGA-3′
	Reverse 5′-CGGCACAAGAATGGATGAAA-3′

GLUT3	Forward 5′-TCCCCAGAGCTTCTTACCTCAC-3′
	Reverse 5′-CAGCAAAAGCCAAGACATTCAC-3′

GLUT8	Forward 5′-CCAAATGGGAACAACTCATCAA-3′
	Reverse 5′-GGGCAAAACCAGCAACAAA-3′

IGFBP1	Forward 5′-TGGCTCGGGCTAGCTGGATG-3′
	Reverse 5′-ACCAGCACCCAGCGGAATCT-3′

IGFBP2	Forward 5′-TGTGACAAGCATGGCTTGTACA-3′
	Reverse 5′-TCTCCACGCTGCCCATTC-3′

IGFBP3	Forward 5′-ATGGTCCCTGTCGTAGAG-3′
	Reverse 5′-ATCCAGGAAGCGGTTGT-3′

IGFBP4	Forward 5′-TGGTGCGTGGACCGCAAGAC′-3′
	Reverse 5′-AGCGATGGGGGCGTCCCATA-3′

IGFBP5	Forward 5′-TGTGCCTCTGGCAGGGGGTA-3′
	Reverse 5′-CAACACAGCCCACGCTTCCG-3′

IGFBP7	Forward 5′-TGTGAAGTCATTGGCATCC-3′
	Reverse 5′-CCTCTCCTTTGGCATTTGA-3′

IR	Forward 5′-CAAACGGTGACCAAGCCTCA-3′
	Reverse 5′-CATCCTGCCCATCAAACTCC-3′

IRS1	Forward 5′-TCCACCACCACCACCATCAC-3′
	Reverse 5′-ACAGCAGCCGCATCCGAAT-3′

MyoD	Forward 5′-CCGCCGATGACTTCTATG-3′
	Reverse 5′-GTTGGTGGTCTTCCTCTTG-3′

MyoG	Forward 5′-AGGCTGAAGAAGGTGAAC-3′
	Reverse 5′-GCTCGATGTACTGGATGG-3′

Mesp1	Forward 5′-GGTCATCACCCTCCTACA-3′
	Reverse 5′-CCATCTCTGCATCCACAA-3′

MYF5	Forward 5′-GAGGAGGAGGCTGAAGAA-3′
	Reverse 5′-CGGCAGGTGATAGTAGTTC-3′

MYF6	Forward 5′-GGAGGAGGCTGAAGAAGA-3′
	Reverse 5′-CTCTCGATGTAGCTGATGG-3′

GATA4	Forward 5′-TCAGACAAGGAAGCGTAAG-3′
	Reverse 5′-ATGGCAGAGACCGAGAAT-3′

GATA6	Forward 5′-CCGACCACTTGCTATGAA-3′
	Reverse 5′-TTGCTACAGTCATCTGAGTT-3′

Nkx2.5	Forward 5′-GACAGAGGAAGAGGAGGAA-3′
	Reverse 5′-CGTTCGCTAGATGGTCTC-3′

GAPDH	Forward 5′-AGAACATCATCCCAGCGT-3′
	Reverse 5′-AGCCTTCACTACCCTCTTG-3′

**Table 2:** Oxygen concentration and the oxygen consumption rate.

Time (min)	1A: O_2_ concentration (nmol/mL)	1A: O_2_ flux per V (pmol/(s∗mL))	1B: O_2_ concentration (nmol/mL)	1B: O_2_ flux per V (pmol/(s∗mL))
0.03	134.4705		222.2083	
0.07	134.361		222.1058	
0.1	134.3131		222.0585	
0.13	134.2618		222.1088	
0.17	134.1943	31.2253	222.157	1.3718
0.2	134.1481	25.9247	222.0753	-5.4236
0.23	134.0865	26.9524	221.9699	6.9917
0.27	134.0053	29.6908	221.9236	24.3313
0.3	133.9386	31.3172	221.8753	32.2136
0.33	133.8958	29.6935	221.8517	14.5802
0.37	133.8531	28.1554	221.8585	13.5949
0.4	133.8078	27.8145	221.8034	15.271
0.43	133.7513	27.2173	221.7768	17.5375
0.47	133.694	26.4491	221.7354	17.44
0.5	133.6496	27.3053	221.6911	14.1902
0.53	133.5888	26.5373	221.6527	14.0926
0.57	133.5316	26.3677	221.6044	15.079
0.6	133.4914	26.0266	221.5699	16.3605
0.63	133.4358	25.6004	221.5187	16.5588
0.67	133.3802	26.457	221.499	14.8846
0.7	133.3238	25.9453	221.4684	14.7868
0.73	133.2365	26.033	221.431	15.2803
0.77	133.1827	26.1198	221.3995	15.7737
0.8	133.1467	25.9497	221.365	15.676
0.83	133.1014	26.4639	221.3246	14.7904
0.87	133.0279	26.2092	221.2931	14.6927
0.9	132.9774	26.2105	221.2497	14.9893
0.93	132.9338	26.1261	221.2143	15.2857
0.97	132.8663	26.0422	221.1739	15.2868
1	132.8064	26.4713	221.1473	14.6963
1.03	132.7414	26.3019	221.1256	14.5984
1.07	132.6687	26.3892	221.0951	14.6976
1.1	132.6345	26.3901	221.0586	14.8956
1.13	132.5969	26.3055	221.0182	14.7981
1.17	132.5524	26.4776	220.9788	14.5035
1.2	132.4772	26.3085	220.9493	14.4057
1.23	132.4361	26.3095	220.9168	14.5051
1.27	132.3746	26.3111	220.8714	14.6047
1.3	132.3147	26.2271	220.836	14.6056
1.33	132.2617	26.3994	220.7926	14.4097
1.37	132.189	26.3157	220.7394	14.411
1.4	132.124	26.4028	220.7089	14.5103
1.43	132.089	26.4037	220.6685	14.7083
1.47	132.024	26.3198	220.6439	14.6104
1.5	131.9581	26.407	220.6064	14.5128
1.53	131.9094	26.4082	220.5749	14.3166
1.57	131.8777	26.409	220.5385	14.3175
1.6	131.8495	26.3242	220.4872	14.4173
1.63	131.7649	26.4118	220.4695	14.4177
1.67	131.6828	26.4994	220.4242	14.5174
1.7	131.6366	26.5005	220.3641	14.5189
1.73	131.5896	26.5017	220.3326	14.5197
1.77	131.5374	26.503	220.3099	14.5202
1.8	131.5041	26.5039	220.2744	14.5211
1.83	131.4545	26.4196	220.2311	14.5222
1.87	131.3698	26.4217	220.2134	14.5227
1.9	131.2988	26.4235	220.1651	14.6224
1.93	131.2518	26.4247	220.1099	14.6238
1.97	131.1979	26.426	220.0469	14.8224
2	131.1304	26.4277	220.0232	14.9215
2.03	131.0833	26.4289	219.9888	14.9223
2.07	131.0269	26.4303	219.9523	15.0217
2.1	130.9645	26.5173	219.909	15.1213
2.13	130.9021	26.5189	219.8597	15.2211
2.17	130.8559	26.5201	219.8272	15.3204
2.2	130.802	26.6069	219.7829	15.3215
2.23	130.7396	26.6085	219.7464	15.421
2.27	130.6797	26.61	219.7228	15.5201
2.3	130.6318	26.6112	219.6735	15.6198
2.33	130.5899	26.6122	219.639	15.7192
2.37	130.5532	26.6131	219.71	15.5204
2.4	130.5147	26.6141	219.71	15.3233
2.43	130.4591	26.53	219.6095	15.1288
2.47	130.3608	26.5324	219.5297	15.1308
2.5	130.3129	26.5336	219.4824	15.132
2.53	130.2658	26.4493	219.441	15.133
2.57	130.2085	26.4507	219.3996	15.1341
2.6	130.1632	26.3664	219.3977	15.0356
2.63	130.1119	26.3676	219.3455	15.0369
2.67	130.0546	26.2836	219.2676	15.0389
2.7	129.9785	26.371	219.2115	15.1388
2.73	129.9281	26.3722	219.1632	15.2385
2.77	129.887	26.3733	219.114	15.4368
2.8	129.8391	26.2889	219.0834	15.536
2.83	129.781	26.2904	219.048	15.6354
2.87	129.7194	26.2919	219.0145	15.6363
2.9	129.6715	26.2931	218.979	15.7357
2.93	129.6159	26.209	218.9317	15.7369
2.97	129.5441	26.2108	218.8736	15.8368
3	129.4774	26.2125	218.8726	15.8369
3.03	129.4099	26.2997	218.8539	15.9358
3.07	129.368	26.3007	218.8224	15.9366
3.1	129.3235	26.3018	218.7642	15.9381
3.13	129.2585	26.3035	218.7248	15.9391
3.17	129.2055	26.2193	218.6795	15.9402
3.2	129.1627	26.2203	218.6342	15.9413
3.23	129.1157	26.2215	218.5977	15.9422
3.27	129.0456	26.2233	218.5652	15.943
3.3	128.9874	26.2247	218.5278	16.0425
3.33	128.9421	26.2259	218.4579	16.1428
3.37	128.9062	26.2268	218.4214	16.2422
3.4	128.8455	26.2283	218.3987	16.2428
3.43	128.7796	26.2299	218.3524	16.3424
3.47	128.7463	26.2308	218.316	16.4418
3.5	128.6693	26.2327	218.2786	16.5413
3.53	128.5992	26.3199	218.2559	16.5419
3.57	128.5308	26.4072	218.2234	16.6412
3.6	128.4803	26.4939	218.18	16.6423
3.63	128.4231	26.5809	218.1288	16.7421
3.67	128.3837	26.5819	218.0855	16.8417
3.7	128.3162	26.5836	218.0559	16.6454
3.73	128.2469	26.5853	218.0185	16.3508
3.77	128.1947	26.5866	217.9643	16.2536
3.8	128.1494	26.6732	217.9436	16.2541
3.83	128.1032	26.6744	217.9298	16.0574
3.87	128.0656	26.6753	217.8875	15.96
3.9	128.0075	26.5913	217.8421	15.9611
3.93	127.9288	26.5932	217.7978	15.7652
3.97	127.8724	26.5947	217.7387	15.6682
4	127.845	26.5953	217.6865	15.768
4.03	127.7877	26.5968	217.6372	15.7692
4.07	127.7099	26.5987	217.6225	15.8681
4.1	127.6654	26.5998	217.6008	15.8686
4.13	127.6081	26.6013	217.5614	15.8696
4.17	127.5363	26.6031	217.5259	15.8705
4.2	127.4713	26.6047	217.4767	15.8717
4.23	127.408	26.6063	217.4402	15.8727
4.27	127.3618	26.6074	217.3969	15.8737
4.3	127.3105	26.6942	217.3565	15.9733
4.33	127.2763	26.6951	217.325	15.9741
4.37	127.2233	26.6964	217.2836	15.9751
4.4	127.1626	26.6979	217.2422	15.8776
4.43	127.1216	26.6989	217.1742	15.8793
4.47	127.054	26.7006	217.1378	15.9787
4.5	126.9813	26.7024	217.0875	15.98
4.53	126.942	26.7034	217.0294	16.08
4.57	126.8941	26.7046	217.0038	16.1791
4.6	126.8342	26.6206	216.9585	16.2788
4.63	126.7932	26.6216	216.8984	16.3788
4.67	126.7145	26.6236	216.8846	16.4776
4.7	126.6461	26.6253	216.858	16.4783
4.73	126.5948	26.5411	216.8235	16.5777
4.77	126.5401	26.5424	216.7762	16.5789
4.8	126.4862	26.5438	216.7457	16.6781
4.83	126.4529	26.4591	216.7033	16.6792
4.87	126.3981	26.4605	216.6708	16.68
4.9	126.3494	26.4617	216.6294	16.681
4.93	126.2955	26.3775	216.5881	16.6821
4.97	126.2314	26.3791	216.5703	16.6825
5	126.1527	26.3811	216.5102	16.684
5.03	126.0963	26.468	216.4748	16.6849
5.07	126.0638	26.4688	216.4482	16.6856
5.1	126.0159	26.47	216.4009	16.6868
5.13	125.9492	26.4717	216.3457	16.6881
5.17	125.9039	26.4728	216.3388	16.5898
5.2	125.844	26.3888	216.4088	16.2925
5.23	125.7901	26.3902	216.3132	16.0979
5.27	125.726	26.3918	216.2078	16.1005
5.3	125.6944	26.3071	216.1861	16.0025
5.33	125.6525	26.2226	216.1142	16.1028
5.37	125.5875	26.1387	216.0659	16.2026
5.4	125.5413	26.1399	215.997	16.3028
5.43	125.4806	26.0559	216.0039	16.2041
5.47	125.4224	25.9718	215.9891	16.106
5.5	125.3745	25.973	215.9359	16.1073
5.53	125.3335	25.974	215.8955	16.0098
5.57	125.2753	25.9755	215.8601	16.0107
5.6	125.2163	25.977	215.8039	16.0121
5.63	125.1462	25.9787	215.7743	15.9143
5.67	125.0855	25.9802	215.7409	15.9151
5.7	125.0393	25.8959	215.6946	15.8178
5.73	124.9889	25.8971	215.6443	15.819
5.77	124.9119	25.8991	215.5921	15.9189
5.8	124.8709	25.9001	215.5665	15.9195
5.83	124.8264	25.9012	215.5192	15.9207
5.87	124.7777	25.9024	215.4818	16.0201
5.9	124.7204	25.8183	215.464	16.1191
5.93	124.6631	25.8198	215.4227	16.1201
5.97	124.5827	25.8218	215.3586	16.2202
6	124.5117	25.9091	215.3468	16.3191
6.03	124.4827	25.9953	215.3212	16.3197
6.07	124.4408	25.9963	215.2867	16.3206
6.1	124.4091	25.9971	215.2256	16.3221
6.13	124.3552	25.9985	215.1675	16.422
6.17	124.3014	25.9143	215.1261	16.4231
6.2	124.2501	25.9156	215.0995	16.4237
6.23	124.1782	25.8319	215.0641	16.5231
6.27	124.1115	25.8335	215.001	16.5247
6.3	124.0619	25.8348	214.9695	16.624
6.33	124.014	25.9215	214.9439	16.6247
6.37	123.9456	26.0087	214.9252	16.6251
6.4	123.8875	26.0102	214.8729	16.6264
6.43	123.8302	26.0116	214.8158	16.6279
6.47	123.7609	26.0989	214.7577	16.7278
6.5	123.7301	26.1851	214.7321	16.7285
6.53	123.684	26.1863	214.7045	16.5321
6.57	123.6215	26.1878	214.672	16.3359
6.6	123.5685	26.2747	214.6276	16.337
6.63	123.5138	26.2761	214.5715	16.3384
6.67	123.4497	26.2777	214.535	16.3393
6.7	123.4009	26.2789	214.4897	16.439
6.73	123.3778	26.1939	214.4395	16.5388
6.77	123.3077	26.1957	214.3794	16.6388
6.8	123.2479	26.1972	214.339	16.7383
6.83	123.2119	26.1126	214.3065	16.7391
6.87	123.1401	26.1144	214.2681	16.8386
6.9	123.1025	26.0298	214.2287	16.8396
6.93	123.0332	26.0315	214.1705	16.9395
6.97	122.987	25.9472	214.1252	17.0392
7	122.9255	25.9487	214.0888	17.0401
7.03	122.875	25.95	214.0799	17.0403
7.07	122.8015	25.9518	214.0504	17.0411
7.1	122.7596	26.0384	214.01	17.0421
7.13	122.7339	25.9535	213.9587	17.1419
7.17	122.663	25.9553	213.8967	17.2419
7.2	122.6134	25.9565	213.8632	17.2428
7.23	122.5544	25.8725	213.8346	17.2435
7.27	122.5013	25.8738	213.8248	17.1452
7.3	122.4304	25.9611	213.7834	17.1462
7.33	122.3731	26.048	213.7528	17.0485
7.37	122.3132	26.0495	213.7213	16.9508
7.4	122.279	26.0504	213.6662	16.8536
7.43	122.2243	26.0518	213.6041	16.8552
7.47	122.1619	25.9678	213.5509	16.8565
7.5	122.0986	25.9694	213.4987	16.9563
7.53	122.0456	25.9707	213.4484	16.9576
7.57	122.0139	25.9715	213.4031	16.9587
7.6	121.9806	25.8868	213.3824	17.0578
7.63	121.9173	25.8029	213.3529	17.0585
7.67	121.8626	25.8043	213.3036	17.0597
7.7	121.8078	25.8056	213.2652	16.9622
7.73	121.7326	25.8075	213.2327	16.963
7.77	121.6873	25.8086	213.213	16.865
7.8	121.6248	25.8957	213.1834	16.7672
7.83	121.5855	25.8967	213.1174	16.7688
7.87	121.547	25.8122	213.0642	16.7702
7.9	121.4957	25.8134	213.0258	16.7711
7.93	121.4495	25.8146	212.9894	16.6735
7.97	121.3803	25.7308	212.949	16.6745
8	121.3161	25.8179	212.9175	16.5768
8.03	121.2648	25.8192	212.9027	16.5772
8.07	121.2092	25.8206	212.8455	16.4801
8.1	121.1742	25.736	212.8081	16.481
8.13	121.1349	25.6514	212.7677	16.5806
8.17	121.0835	25.5672	212.7234	16.5817
8.2	121.0117	25.569	212.6643	16.5831
8.23	120.9604	25.4848	212.614	16.6829
8.27	120.8852	25.5722	212.5707	16.7825
8.3	120.8227	25.5737	212.5559	16.7829
8.33	120.7919	25.5745	212.5165	16.8824
8.37	120.7458	25.4901	212.4958	16.7844
8.4	120.6979	25.4913	212.4791	16.6863
8.43	120.6346	25.4929	212.4111	16.688
8.47	120.5679	25.4946	212.4022	16.5897
8.5	120.5183	25.4958	212.4633	16.3911
8.53	120.4773	25.4968	212.3402	16.2957
8.57	120.4277	25.4126	212.2249	16.3971
8.6	120.355	25.4144	212.1825	16.3982
8.63	120.3105	25.4155	212.1116	16.4984
8.67	120.2857	25.4161	212.0653	16.4996
8.7	120.237	25.4173	212.0722	16.4009
8.73	120.166	25.4191	212.0702	16.3025
8.77	120.1292	25.3345	212.018	16.3038
8.8	120.0754	25.3359	211.955	16.3053
8.83	119.9967	25.4233	211.9087	16.405
8.87	119.9497	25.4245	211.8781	16.5043
8.9	119.9009	25.4257	211.8998	16.4052
8.93	119.8265	25.4276	211.8703	16.3075
8.97	119.7752	25.4289	211.7816	16.3097
9	119.7059	25.5161	211.7107	16.41
9.03	119.6717	25.517	211.6584	16.5098
9.07	119.617	25.6039	211.5944	16.6099
9.1	119.5503	25.6055	211.5461	16.7096
9.13	119.4981	25.6923	211.5245	16.8087
9.17	119.434	25.7795	211.4782	16.8098
9.2	119.3853	25.7807	211.4289	17.0081
9.23	119.3348	25.8675	211.3816	17.1078
9.27	119.2698	25.8691	211.3787	17.2064
9.3	119.2356	25.8699	211.3639	17.2067
9.33	119.2006	25.8708	211.3087	17.2081
9.37	119.1458	25.8722	211.354	17.01
9.4	119.1022	25.8733	211.3797	16.8123
9.43	119.0672	25.7886	211.2358	16.7174
9.47	119.0167	25.7044	211.1373	16.7198
9.5	118.9432	25.6207	211.0743	16.8199
9.53	118.8842	25.6222	211.0181	17.0183
9.57	118.8551	25.5374	211.0181	17.1169
9.6	118.8012	25.5387	210.9698	17.2166
9.63	118.726	25.5406	210.9294	17.3161
9.67	118.6618	25.6277	210.8605	17.4163
9.7	118.6148	25.5434	210.8221	17.5158
9.73	118.5567	25.5449	210.7679	17.5172
9.77	118.5036	25.5462	210.7699	17.5171
9.8	118.4224	25.6337	210.8802	17.2188
9.83	118.3933	25.72	210.7452	16.9267
9.87	118.3591	25.6353	210.6369	16.8309
9.9	118.2958	25.6369	210.5807	16.9308
9.93	118.2266	25.7241	210.5226	17.0307
9.97	118.1958	25.7249	210.4714	17.229
10	118.1376	25.6408	210.4832	17.4258
10.03	118.0855	25.6422	210.428	17.4272
10.07	118.0299	25.558	210.3935	17.428
10.1	117.994	25.4734	210.3591	17.4289
10.13	117.9307	25.3895	210.3059	17.4302
10.17	117.8666	25.3911	210.294	17.4305
10.2	117.8016	25.3927	210.3728	17.2315
10.23	117.7708	25.3935	210.2822	16.9382
10.27	117.7272	25.3946	210.166	16.8426
10.3	117.6562	25.3963	210.1246	16.7451
10.33	117.5972	25.3978	210.0576	16.8453
10.37	117.5716	25.3985	210.0162	16.9449
10.4	117.51	25.4	210.0113	16.945
10.43	117.4536	25.4014	209.968	17.0446
10.47	117.4193	25.4023	209.9088	17.1446
10.5	117.3783	25.4033	209.8714	17.244
10.53	117.2954	25.4054	209.8419	17.2448
10.57	117.2355	25.4924	209.7877	17.4432
10.6	117.1962	25.5789	209.7512	17.4441
10.63	117.1551	25.5799	209.7059	17.4452
10.67	117.1004	25.4958	209.6665	17.4462
10.7	117.0474	25.4971	209.6143	17.349
10.73	116.9721	25.499	209.6833	16.9532
10.77	116.9345	25.4999	209.6232	16.6592
10.8	116.9046	25.3296	209.4882	16.7611
10.83	116.8396	25.3313	209.4616	16.8602
10.87	116.7891	25.247	209.3897	16.9605
10.9	116.7464	25.1626	209.3463	17.0601
10.93	116.6891	25.0785	209.3286	17.1591
10.97	116.6395	24.9942	209.3444	17.0602
11	116.5856	24.9956	209.2793	17.1603
11.03	116.5377	24.9967	209.2153	17.2604
11.07	116.4676	24.9985	209.1789	17.3599
11.1	116.4163	24.9998	209.1345	17.361
11.13	116.3522	25.0869	209.0971	17.1649
11.17	116.3248	25.0021	209.1651	16.8676
11.2	116.2735	25.0034	209.0853	16.7711
11.23	116.2059	25.005	208.969	16.8725
11.27	116.1444	25.0921	208.9582	16.8728
11.3	116.0871	25.0935	208.9119	17.071
11.33	116.0229	25.1806	208.8528	17.0725
11.37	115.9836	25.1816	208.837	17.0729
11.4	115.946	25.1826	208.7878	17.1726
11.43	115.8827	25.1841	208.7444	17.1737
11.47	115.8254	25.1856	208.6873	17.2736
11.5	115.7809	25.2722	208.6548	17.2745
11.53	115.7219	25.3592	208.6193	17.0783
11.57	115.6792	25.3603	208.5819	16.9807
11.6	115.6125	25.3619	208.6223	16.7827
11.63	115.5646	25.4486	208.637	16.5853
11.67	115.5295	25.4495	208.5366	16.4893
11.7	115.4825	25.4507	208.4577	16.4913
11.73	115.4235	25.4522	208.442	16.3932
11.77	115.3713	25.4535	208.3848	16.2961
11.8	115.3294	25.369	208.311	16.3964
11.83	115.2867	25.2846	208.3159	16.2978
11.87	115.2268	25.2861	208.2676	16.299
11.9	115.1704	25.2875	208.2243	16.3001
11.93	115.1088	25.3745	208.1819	16.3011
11.97	115.0558	25.2903	208.1504	16.3019
12	114.9959	25.2918	208.0982	16.3032
12.03	114.9549	25.2929	208.0578	16.3042
12.07	114.8967	25.3798	208.0282	16.108
12.1	114.8258	25.3816	208.1248	15.7115
12.13	114.7727	25.3829	208.0134	15.7143
12.17	114.7326	25.4694	207.8992	15.7171
12.2	114.6787	25.4708	207.8736	15.8163
12.23	114.6334	25.3864	207.8223	15.9161
12.27	114.6043	25.3016	207.7583	16.0162
12.3	114.553	25.3029	207.7741	16.0158
12.33	114.4846	25.2191	207.7534	15.9178
12.37	114.4127	25.2209	207.6982	15.9192
12.4	114.3537	25.2224	207.6588	15.9202
12.43	114.3093	25.309	207.6095	16.0199
12.47	114.2426	25.3107	207.5268	16.1205
12.5	114.2015	25.3117	207.5051	16.0225
12.53	114.1733	25.3124	207.5603	15.8241
12.57	114.1314	25.2279	207.5573	15.7257
12.6	114.0604	25.2297	207.4293	15.7289
12.63	113.9963	25.2313	207.3593	15.7306
12.67	113.9535	25.3179	207.2973	15.9292
12.7	113.9056	25.3191	207.247	16.029
12.73	113.8509	25.3205	207.2224	16.1281
12.77	113.8184	25.3213	207.246	16.226
12.8	113.7663	25.2371	207.1948	16.2273
12.83	113.7141	25.2384	207.1377	16.3273
12.87	113.6551	25.2398	207.1002	16.4267
12.9	113.5833	25.2416	207.0451	16.6251
12.93	113.5285	25.3285	206.9948	16.6264
12.97	113.4841	25.3296	206.9603	16.4302
13	113.4413	25.3307	206.9298	16.431
13.03	113.3883	25.332	206.8579	16.5313
13.07	113.3327	25.3334	206.8756	16.5308
13.1	113.2848	25.3346	206.9396	16.3322
13.13	113.2258	25.2506	206.8372	16.3348
13.17	113.1839	25.2516	206.7466	16.337
13.2	113.1506	25.1669	206.716	16.3378
13.23	113.0719	25.0834	206.651	16.3394
13.27	113.0155	25.0848	206.5899	16.538
13.3	112.9573	25.0863	206.5939	16.5379
13.33	112.918	25.0872	206.5535	16.5389
13.37	112.8684	25.0885	206.517	16.6383
13.4	112.8025	25.0901	206.4658	16.6396
13.43	112.7624	25.1766	206.4106	16.4439
13.47	112.7145	25.1778	206.3643	16.4451
13.5	112.6572	25.1793	206.3121	16.5449
13.53	112.5965	25.1808	206.2688	16.6445
13.57	112.5306	25.268	206.2363	16.7438
13.6	112.493	25.1834	206.2116	16.843
13.63	112.4554	25.1843	206.1821	16.8437
13.67	112.3998	25.1002	206.1545	16.8444
13.7	112.3425	25.1871	206.122	16.7467
13.73	112.2963	25.1883	206.0875	16.7476
13.77	112.2279	25.2755	206.053	16.7484
13.8	112.1638	25.3626	206.0176	16.7493
13.83	112.1219	25.3637	205.988	16.75
13.87	112.0791	25.3648	205.925	16.6531
13.9	112.0124	25.3664	205.9171	16.3578
13.93	111.962	25.4532	205.986	16.0605
13.97	111.9286	25.454	205.8836	15.866
14	111.8696	25.4555	205.7969	15.9667
14.03	111.826	25.4566	205.7732	15.9673
14.07	111.7644	25.4581	205.7102	15.9689
14.1	111.7251	25.3736	205.659	15.9702
14.13	111.6738	25.3749	205.6639	15.8715
14.17	111.6105	25.291	205.5861	15.8735
14.2	111.5566	25.2923	205.522	15.8751
14.23	111.4865	25.3796	205.4826	15.9746
14.27	111.4455	25.3806	205.4521	16.0739
14.3	111.4164	25.3813	205.4186	16.0747
14.33	111.36	25.3827	205.4018	16.0751
14.37	111.3009	25.3842	205.3506	16.1749
14.4	111.2325	25.3859	205.3201	16.1757
14.43	111.1906	25.387	205.2836	15.9795
14.47	111.1479	25.388	205.3152	15.6832
14.5	111.0923	25.3039	205.324	15.486
14.53	111.0324	25.3054	205.2265	15.3899
14.57	110.9657	25.3071	205.1408	15.392
14.6	110.9332	25.3079	205.0945	15.4917
14.63	110.876	25.3093	205.0373	15.4931
14.67	110.811	25.3965	204.992	15.4943
14.7	110.7622	25.3977	204.9782	15.4946
14.73	110.7118	25.3989	204.9142	15.5947
14.77	110.6485	25.4005	204.8728	15.6943
14.8	110.6066	25.4016	204.8482	15.7934
14.83	110.5844	25.4021	204.8029	15.8931
14.87	110.5262	25.3181	204.7418	16.0916
14.9	110.4715	25.3194	204.7162	16.2893
14.93	110.4108	25.3209	204.6926	16.3884
14.97	110.3586	25.3223	204.6591	16.4877
15	110.297	25.3238	204.6118	16.5874
15.03	110.2474	25.325	204.5763	16.5883
15.07	110.203	25.3261	204.5605	16.5887
15.1	110.1371	25.3278	204.5162	16.5898
15.13	110.079	25.4148	204.4719	16.6894
15.17	110.0328	25.4159	204.4098	16.691
15.2	109.9926	25.4169	204.3803	16.7902
15.23	109.9302	25.4185	204.3744	16.6919
15.27	109.8883	25.505	204.4561	16.2958
15.3	109.8609	25.4202	204.3576	16.0027
15.33	109.8122	25.3359	204.2778	15.9062
15.37	109.7386	25.3378	204.2266	15.9075
15.4	109.6762	25.3393	204.1566	15.9092
15.43	109.63	25.255	204.1133	16.0088
15.47	109.5796	25.2562	204.1172	15.8117
15.5	109.5377	25.2573	204.0798	15.8126
15.53	109.4975	25.1728	204.0148	15.9128
15.57	109.4633	25.1736	203.9606	16.0126
15.6	109.3966	25.0898	203.933	16.1118
15.63	109.3188	25.0917	203.8966	16.1127
15.67	109.264	25.0931	203.867	16.1135
15.7	109.2221	25.0086	203.8246	16.2131
15.73	109.1512	25.0959	203.7872	16.214
15.77	109.1204	25.0967	203.7557	16.2148
15.8	109.0853	25.012	203.7251	16.117
15.83	109.0297	24.9279	203.6769	15.8227
15.87	108.9853	24.929	203.6355	15.7252
15.9	108.9297	24.9304	203.5941	15.7262
15.93	108.8775	24.8462	203.5567	15.7272
15.97	108.8151	24.8478	203.5193	15.7281
16	108.7647	24.849	203.4917	15.8273
16.03	108.7245	24.85	203.4651	15.7295
16.07	108.6757	24.8512	203.4207	15.7306
16.1	108.6364	24.8522	203.3498	15.8309
16.13	108.5765	24.8537	203.2897	15.9309
16.17	108.5167	24.7697	203.272	16.0298
16.2	108.4594	24.7711	203.2375	16.1292
16.23	108.3978	24.7727	203.1922	16.2289
16.27	108.3499	24.7739	203.1518	16.3284
16.3	108.3037	24.775	203.137	16.3288
16.33	108.2576	24.7762	203.1055	16.4281
16.37	108.1986	24.7777	203.0612	16.4292
16.4	108.1558	24.7787	203.0011	16.4307
16.43	108.1131	24.7798	202.9705	16.5299
16.47	108.0446	24.867	202.9508	16.5304
16.5	107.9856	24.8685	202.9173	16.5313
16.53	107.9352	24.9553	202.8809	16.6307
16.57	107.8805	25.0422	202.8375	16.5333
16.6	107.8497	25.0429	202.7804	16.3377
16.63	107.8086	24.9584	202.7489	16.2399
16.67	107.7633	24.8741	202.7193	16.1422
16.7	107.7094	24.8754	202.6908	16.0444
16.73	107.6436	24.8771	202.6484	16.1439
16.77	107.6111	24.8779	202.6307	16.0459
16.8	107.5435	24.8796	202.6031	15.948
16.83	107.4939	24.8808	202.5489	15.8509
16.87	107.4486	24.8819	202.5046	15.852
16.9	107.3905	24.7979	202.4721	15.8528
16.93	107.3152	24.7998	202.409	15.8544
16.97	107.2827	24.8861	202.3755	15.8552
17	107.2391	24.8872	202.3469	15.8559
17.03	107.1724	24.8888	202.3184	15.8567
17.07	107.1126	24.9758	202.275	15.7592
17.1	107.0561	25.0628	202.2445	15.76
17.13	106.9997	25.0642	202.1962	15.7612
17.17	106.9544	25.1508	202.1489	15.7624
17.2	106.9202	25.1517	202.1135	15.6647
17.23	106.8783	25.0672	202.1834	15.466
17.27	106.8158	25.0688	202.1263	15.3689
17.3	106.7577	25.1557	202.0248	15.3714
17.33	106.7184	25.1567	201.9992	15.2736
17.37	106.6611	25.1582	201.946	15.1764
17.4	106.5995	25.1597	201.8967	15.1776
17.43	106.5499	25.0754	201.8958	15.1776
17.47	106.5011	25.0766	201.8524	15.1787
17.5	106.4687	25.0775	201.8022	15.18
17.53	106.4208	25.0786	201.7657	15.1809
17.57	106.3541	25.0803	201.7174	15.2806
17.6	106.3173	25.0812	201.6593	15.3806
17.63	106.2609	25.0826	201.617	15.4801
17.67	106.1907	25.0844	201.5825	15.481
17.7	106.1454	25.0855	201.547	15.5804
17.73	106.0924	25.0869	201.5155	15.5812
17.77	106.0445	25.0881	201.4692	15.6809
17.8	105.9975	25.0892	201.4396	15.7801
17.83	105.9376	25.1762	201.3845	15.7815
17.87	105.8923	25.1774	201.4436	15.6815
17.9	105.8376	25.1787	201.4495	15.4843
17.93	105.788	25.18	201.3254	15.4874
17.97	105.7521	25.1809	201.2899	15.4883
18	105.6973	25.0967	201.219	15.5886
18.03	105.6221	25.0986	201.151	15.6888
18.07	105.5785	25.0997	201.148	15.7874
18.1	105.5417	25.0151	201.1106	15.7883
18.13	105.475	25.0168	201.0584	15.7896
18.17	105.4186	25.0182	200.9953	15.8897
18.2	105.3724	25.0193	200.9648	15.8905
18.23	105.3262	25.0205	200.9313	15.9898
18.27	105.2826	25.0216	200.9047	16.089
18.3	105.2287	24.9374	200.8781	16.0897
18.33	105.168	24.9389	200.9707	15.8903
18.37	105.1201	24.9401	200.8722	15.8928
18.4	105.074	24.9413	200.7747	15.8952
18.43	105.0363	24.9422	200.7491	15.8959
18.47	104.973	25.0293	200.6919	15.8973
18.5	104.9235	25.0306	200.6338	16.0958
18.53	104.8781	25.0317	200.6141	16.0963
18.57	104.8123	24.9478	200.5757	15.9987
18.6	104.7413	25.0351	200.5382	15.9012
18.63	104.708	25.036	200.5028	15.902
18.67	104.6703	25.0369	200.4446	15.9035
18.7	104.631	24.9524	200.4141	16.0028
18.73	104.5805	24.9536	200.3727	16.0038
18.77	104.5181	24.9552	200.3323	16.0048
18.8	104.4651	24.9565	200.3195	16.0051
18.83	104.4044	24.958	200.3796	15.8066
18.87	104.3625	24.9591	200.2663	15.7109
18.9	104.3197	24.8746	200.1777	15.8117
18.93	104.2607	24.8761	200.1461	15.8124
18.97	104.2154	24.8772	200.1077	15.9119
19	104.1701	24.7929	200.0664	16.0115
19.03	104.1111	24.7943	200.0752	16.0112
19.07	104.0623	24.7956	200.0181	16.1112
19.1	104.0222	24.7966	199.9629	16.1126
19.13	103.9862	24.712	199.9215	16.2121
19.17	103.9221	24.7136	199.8821	16.3116
19.2	103.8853	24.629	199.8792	16.2132
19.23	103.8263	24.6304	199.823	16.0175
19.27	103.763	24.632	199.7767	15.9202
19.3	103.7194	24.5476	199.8575	15.7211
19.33	103.6767	24.4632	199.7807	15.526
19.37	103.6245	24.4645	199.6605	15.6276
19.4	103.5476	24.5519	199.6437	15.628
19.43	103.5022	24.553	199.5817	15.728
19.47	103.4595	24.5541	199.5324	15.8278
19.5	103.4107	24.5553	199.5166	15.9267
19.53	103.3654	24.5564	199.4851	16.026
19.57	103.3218	24.5575	199.4585	16.0267
19.6	103.2722	24.4733	199.4103	16.1264
19.63	103.2064	24.4749	199.3679	16.1274
19.67	103.1653	24.4759	199.3068	15.9319
19.7	103.12	24.4771	199.2645	15.933
19.73	103.0618	24.4785	199.2507	15.9333
19.77	103.0063	24.4799	199.3393	15.6356
19.8	102.9524	24.4813	199.2241	15.6385
19.83	102.9028	24.4825	199.1285	15.6409
19.87	102.8412	24.5696	199.0999	15.7401
19.9	102.8045	24.5705	199.0379	15.8402
19.93	102.7514	24.6573	198.9975	15.8412
19.97	102.6916	24.6588	198.9935	15.9398
20	102.6463	24.7455	198.9512	15.9408
20.03	102.5941	24.7468	198.8931	16.0408
20.07	102.5342	24.7483	198.8428	16.1406
20.1	102.4821	24.7496	198.8133	16.2398
20.13	102.4573	24.7502	198.7857	16.339
20.17	102.4111	24.7513	198.7561	16.1427
20.2	102.3624	24.7525	198.697	16.1442
20.23	102.2982	24.7542	198.6813	16.1446
20.27	102.2452	24.841	198.7532	16.0443
20.3	102.2127	24.8418	198.6172	16.0477
20.33	102.1597	24.7576	198.5364	16.1482
20.37	102.087	24.8449	198.5	16.2477
20.4	102.0477	24.8459	198.4488	16.3474
20.43	101.9964	24.8472	198.4094	16.4469
20.47	101.9553	24.7627	198.4143	16.4468
20.5	101.9134	24.7638	198.3493	16.547
20.53	101.8647	24.6795	198.3089	16.548
20.57	101.798	24.6811	198.2586	16.6477
20.6	101.7338	24.6827	198.2271	16.747
20.63	101.6817	24.6841	198.1926	16.6494
20.67	101.6372	24.6852	198.1808	16.4527
20.7	101.5928	24.6863	198.1335	16.4538
20.73	101.5508	24.6873	198.1197	16.4542
20.77	101.4953	24.6887	198.1936	16.2553
20.8	101.4431	24.69	198.0685	16.1599
20.83	101.3952	24.6912	197.9907	16.1619
20.87	101.3499	24.6923	197.968	16.1624
20.9	101.296	24.6937	197.901	16.1641
20.93	101.2285	24.6954	197.8577	16.1652
20.97	101.1797	24.6966	197.8616	16.1651
21	101.131	24.6978	197.8104	16.1664
21.03	101.0771	24.6992	197.7444	16.2665
21.07	101.0275	24.7004	197.6823	16.3666
21.1	100.9796	24.7016	197.6518	16.1703
21.13	100.9437	24.7025	197.64	16.0721
21.17	100.9027	24.618	197.6114	16.0728
21.2	100.8556	24.6192	197.571	16.0739
21.23	100.8095	24.6203	197.5286	16.0749
21.27	100.759	24.5361	197.5996	15.9746
21.3	100.7017	24.5375	197.505	15.78
21.33	100.6427	24.539	197.4085	15.8809
21.37	100.5948	24.5402	197.373	15.8818
21.4	100.5392	24.6271	197.3257	15.9815
21.43	100.4896	24.6283	197.2981	15.9822
21.47	100.4452	24.6295	197.2804	15.9826
21.5	100.3879	24.6309	197.2311	15.9838
21.53	100.3468	24.5464	197.1917	15.9848
21.57	100.2964	24.5477	197.1474	16.0844
21.6	100.2357	24.6347	197.1178	15.7896
21.63	100.1818	24.5505	197.0804	15.7906
21.67	100.1219	24.6375	197.0607	15.7911
21.7	100.0886	24.6384	197.1238	15.5925
21.73	100.039	24.6396	197.0646	15.4954
21.77	99.9937	24.6407	196.9642	15.4979
21.8	99.9338	24.6422	196.9326	15.4987
21.83	99.8817	24.558	196.8568	15.5006
21.87	99.8415	24.559	196.8016	15.6005
21.9	99.7902	24.5603	196.8026	15.699
21.93	99.7397	24.5616	196.7612	15.7986
21.97	99.6961	24.5627	196.7208	15.7996
22	99.6294	24.5643	196.6834	15.8005
22.03	99.5918	24.5653	196.6351	15.9002
22.07	99.5405	24.5666	196.5928	15.9013
22.1	99.4909	24.5678	196.5691	15.7048
22.13	99.4396	24.5691	196.5238	15.6075
22.17	99.3865	24.5704	196.5021	15.608

## Data Availability

The data used to support the findings of this study are available from the corresponding author upon request.

## Abbreviations

IGF1: Insulin-like growth factor 1
IGFs: Insulin-like growth factors
IGF1R: Insulin-like growth factor 1 receptor
IGF2: Insulin-like growth factor 2
GH: Growth hormone
BMP: Bone morphogenic protein
FGF4: Fibroblast growth factor 4
IR: Insulin receptor
IGFR: Insulin-like growth factor receptor
IRR: Insulin receptor-related receptor
TK: Tyrosine kinase
IRS: Insulin receptor substrate
PI3K: Phosphatidylinositol 3-kinase
VEGF: Vascular endothelial growth factor
ROS: Reactive oxygen species
H_2_O_2_: Hydrogen peroxide
GSH: Glutathione
GSH-Px: Glutathione peroxidase
CAT: Catalase
MDA: Malondialdehyde
iNOS: Induced nitric oxide synthase
SOD: Superoxide dismutase
T-AOC: Total antioxidant capability
IGFBP1: Insulin-like growth factor-binding protein 1
IGFBP2: Insulin-like growth factor-binding protein 2
IGFBP3: Insulin-like growth factor-binding protein 3
IGFBP4: Insulin-like growth factor-binding protein 4
IGFBP5: Insulin-like growth factor-binding protein 5
IGFBP7: Insulin-like growth factor-binding protein 7
GLUT1: Glucose transporters 1
GLUT3: Glucose transporters 3
GLUT8: Glucose transporters 8
Akt: Threonine-protein kinase
P-Akt: Phosphorylation-threonine-protein kinase
FOXO: Forkhead box protein
P-FOXO: Phosphorylation-forkhead box protein
JNK: c-Jun N-terminal kinase
P-JNK: Phosphorylation-c-Jun N-terminal kinase
GATA4: GATA-binding protein 4
GATA6: GATA-binding protein 6
Nkx2.5: NK2 homeobox 5
AMPK: AMP-activated protein kinase
MyoG: Myogenin
Mesp1: Cardiac transcription factor mesoderm posterior 1
MYF5: Myogenic factor 5
MYF6: Myogenic factor 6
O2^−^: Superoxide anion
GSSH: Oxidized glutathione
ATP: Adenosine triphosphate
IGFBPs: Insulin-like growth factor-binding proteins
MRFs: Myogenic regulatory factors
MAPK: Mitogen-activated protein kinase
Nrf2: Nuclear factor erythroid 2-related factor 2
PBS: Phosphate-buffered solution
GAPDH: Glyceraldehyde-3-phosphate dehydrogenase.

## Supplementary Materials

Supplementary Materials The graphical abstract of the entire manuscript.
