# Supplementary figures and images for: IGF1 Knockdown Hinders Myocardial Development through Energy Metabolism Dysfunction Caused by ROS-Dependent FOXO Activation in the Chicken Heart
Source: Oxid Med Cell Longev. 2019 Dec 24;2019:7838754. doi: 10.1155/2019/7838754 (PMC6948330; doi:10.1155/2019/7838754)

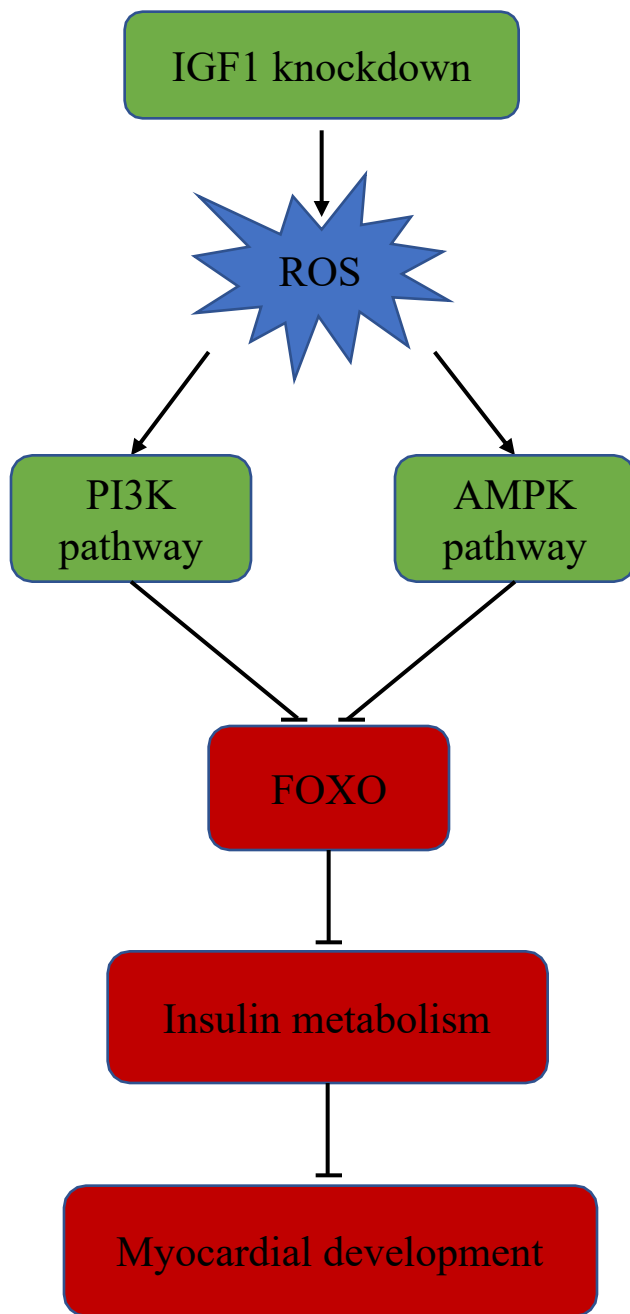

Supplement: Supplementary Materials — The graphical abstract of the entire manuscript. [file 7838754.f1.pdf]
